# Ultrathin inorganic molecular nanowire based on polyoxometalates

**DOI:** 10.1038/ncomms8731

**Published:** 2015-07-03

**Authors:** Zhenxin Zhang, Toru Murayama, Masahiro Sadakane, Hiroko Ariga, Nobuhiro Yasuda, Norihito Sakaguchi, Kiyotaka Asakura, Wataru Ueda

**Affiliations:** 1 Catalysis Research Center, Hokkaido University, N-21, W-10, Kita-ku, Sapporo 001-0021, Japan; 2 Department of Applied Chemistry, Graduate School of Engineering, Hiroshima University, 1-4-1 Kagamiyama, Higashi Hiroshima 739-8527, Japan; 3 JST, PRESTO, 4-1-8 Honcho, Kawaguchi, Saitama, 332-0012, Japan; 4 Japan Synchrotron Radiation Research Institute/SPring-8, 1-1-1 Kouto, Sayocho, Sayogun, Hyogo 679-5198, Japan; 5 High Voltage Electron Microscope Laboratory, Center for Advanced Research of Energy and Materials, Faculty of Engineering, Hokkaido University, Sapporo 060-8626, Japan; 6 Department of Material and Life Chemistry, Faculty of Engineering, Kanagawa University, Rokkakubashi, Kanagawa-ku, Yokohama-shi, Kanagawa 221-8686, Japan

## Abstract

The development of metal oxide-based molecular wires is important for fundamental research and potential practical applications. However, examples of these materials are rare. Here we report an all-inorganic transition metal oxide molecular wire prepared by disassembly of larger crystals. The wires are comprised of molybdenum(VI) with either tellurium(IV) or selenium(IV): {(NH_4_)_2_[XMo_6_O_21_]}_*n*_ (X=tellurium(IV) or selenium(IV)). The ultrathin molecular nanowires with widths of 1.2 nm grow to micrometre-scale crystals and are characterized by single-crystal X-ray analysis, Rietveld analysis, scanning electron microscopy, X-ray photoelectron spectroscopy, ultraviolet–visible spectroscopy, thermal analysis and elemental analysis. The crystals can be disassembled into individual molecular wires through cation exchange and subsequent ultrasound treatment, as visualized by atomic force microscopy and transmission electron microscopy. The ultrathin molecular wire-based material exhibits high activity as an acid catalyst, and the band gap of the molecular wire-based crystal is tunable by heat treatment.

## Abstract (web summary)

Molecular nanowires, one-dimensional materials grown by repeating a molecular unit along a single axis, are fundamentally and technologically interesting materials. Here, the authors report a rare inorganic molecular nanowire comprised of polyoxometalates, and study its catalytic and electronic properties.

## Graphical abstract

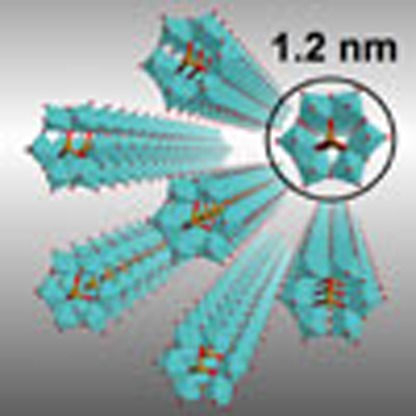

Nanowires, in which two dimensions of the materials are on a scale of tens of nanometres or less and the length of the remaining dimension can be increased without confinement, have been attracting attention because of their large surface area and quantum mechanical effects that result in unique material properties. Different compounds, such as metallic compounds^1^, semiconducting compounds^2^,^3^,^4^,^5^,^6^,^7^,^8^,^9^,^10^, metal oxides^11^,^12^,^13^ and organic polymers^14^,^15^,^16^,^17^, can be grown to form nanowires for use in functional materials and devices that have been successfully applied as sensors^13^, transistors^2^,^5^, semiconductors^3^, photonics devices^7^,^11^ and solar cells^4^,^6^.

Among the various types of nanowires, molecular wires, which grow by repeating a single molecular unit along a certain axis, have garnered much interest. The most common type of molecular wire is an organic or organometallic polymer^14^,^15^,^16^,^17^, which has been widely applied in nanotechnology, semiconductors, electrochemistry and cell biology. A more interesting material is a molecular wire with an all-inorganic composition; inorganic compounds have various advantages over organic molecular wires, including stable structures, tunable chemical compositions and tunable properties. However, all-inorganic molecular wires are rare, leaving a field that is full of challenges. One example of an inorganic molecular wire is the Mo_6_S_9-*x*_I_*x*_ molecular wire^18^,^19^, which was assembled with molecular units of Mo_6_S_9-*x*_I_*x*_ along its *c* axis to form a nanowire. The material exhibits excellent electron transport, magnetic, mechanical, tribological and optical properties and has been applied in chemisensors, biosensors, field emission devices, composites, lubricants, nonlinear optical limiting materials, Li batteries and molecular-scale connectors for molecular electronics.

Assembly of transition metal–oxygen octahedral building blocks is an attractive approach to form nanostructured materials^20^. Both zero-dimensional molecular nanodots, which are known as polyoxometalates (POMs)^21^,^22^, and two-dimensional molecular nanosheets^23^,^24^ can be obtained by this approach. However, no isolated molecular nanowire assembled with transition metal oxide octahedra has been reported to date.

POMs are ideal subunits for constructing one-dimensional (1D) metal oxides that are based on transition metal–oxygen octahedra, and a few examples of all-inorganic POM-based chain structures in crystals have been reported^25^,^26^,^27^. However, isolation of individual ultrathin molecular wires that are based on transition metal oxides has not yet been realized to the best of our knowledge.

Herein, we report an isolable transition metal oxide-based molecular wire that is formed by repeating a hexagonal molecular unit of [X^IV^Mo^VI^_6_O_21_]^2−^ along its *c* axis, where X=Te or Se and denoted as Mo–Te oxide and Mo–Se oxide, respectively. These molecular wires assemble in a hexagonal manner on interaction with water and ammonium cations to form crystals, and the molecular wires are isolable from the crystal. The ultrathin molecular wire-based material acts as an active acid catalyst, and the band gap of the molecular wire-based crystal is easily altered, indicating its potential application in catalysis and electronic devices^28^.

## Results

### Material synthesis and characterization

Crystalline transition metal oxide molecular wires were synthesized using a hydrothermal method. The starting materials contained (NH_4_)_6_Mo_7_O_24_·4H_2_O as a source of Mo, and Se^IV^ or Te^IV^ ions were assembled into the materials, forming Mo–Se oxide or Mo–Te oxide, respectively. The Se or Te ions with IV oxidation state were essential in obtaining these samples. Mo–Se oxide was easily obtained via hydrothermal synthesis of (NH_4_)_6_Mo_7_O_24_·4H_2_O and a Se^IV^O_2_ solution. For Mo–Te oxide, soluble Te^VI^(OH)_6_ was used with a reducing agent (VOSO_4_·5H_2_O) to form Te^IV^ ions. X-ray photoelectron spectroscopy (XPS) with curve fitting (Supplementary Fig. 1a–d) was used to confirm that the Mo, Te and Se ions in both materials were present as Mo^VI^, Te^IV^ and Se^IV^, respectively. Ultraviolet–visible (ultraviolet–vis) spectra of Mo–Te oxide and Mo–Se oxide are presented in Supplementary Fig. 2. No absorption was detected over a range of 500–600 nm, which was attributed to Mo^V^ and confirmed that the Mo ions in the materials were present as Mo^VI^. Elemental analysis, XPS and energy dispersive X-ray spectroscopy analysis confirmed that there was no vanadium present in the Mo–Te oxide (Supplementary Fig. 3); vanadium was therefore not a building block of the material and only acted as a reducing agent to reduce Te^VI^(OH)_6_ to Te^IV^ ion. Elemental analysis of Mo–Te oxide and Mo–Se oxide revealed that the ratio of Mo:Te:NH_4_^+^:H_2_O and Mo:Se:NH_4_^+^:H_2_O was 6:1:2:3 and 6:1:2:2, respectively.

**Figure 1:**
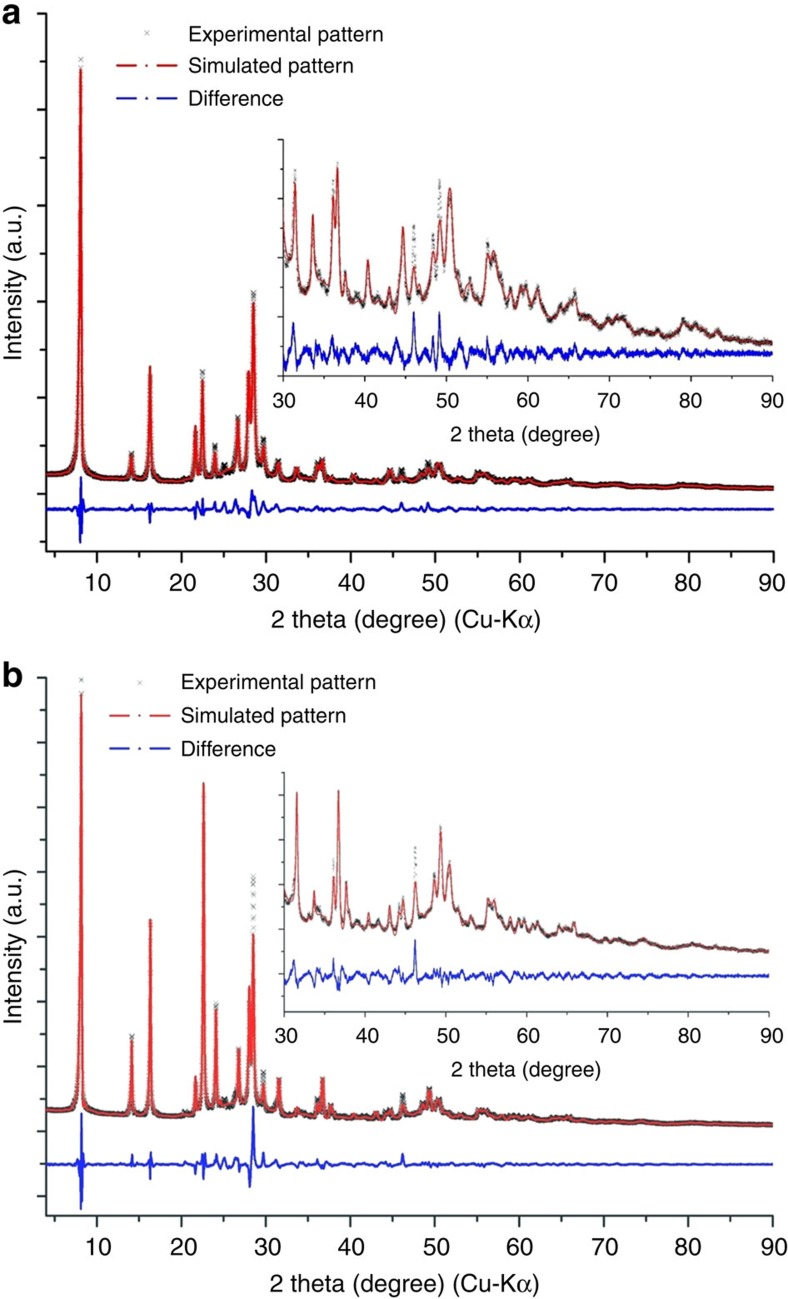
Comparison of the experimental XRD patterns with the simulated XRD patterns using the Rietveld method. (**a**) Mo–Te oxide, *R*_wp_=7.06% and (**b**) Mo–Se oxide, *R*_wp_=7.49%. Insert: magnification of a high-angle area.

Powder X-ray diffraction (XRD) patterns of Mo–Te oxide and Mo–Se oxide are shown in Fig. 1 and Supplementary Fig. 4. The powder XRD peaks of Mo–Te oxide and Mo–Se oxide could be indexed by a trigonal cell with lattice parameters of *a*=12.48 Å and *c*=3.94 Å for Mo–Te oxide and *a*=12.51 Å, *c*=3.93 Å for Mo–Se oxide (Supplementary Table 1). Similar unit cells indicated that the structures of the two materials were similar. The Fourier transform infrared (FTIR) spectra (Supplementary Fig. 4) of Mo–Te oxide and Mo–Se oxide were similar, indicating that water (1,620 cm^−1^) and NH_4_^+^ (1,400 cm^−1^) were present in the materials and that the molecular structures were the same. The presence of water and NH_4_^+^ were also confirmed by thermogravimetric-differential thermal analysis (TG-DTA) (Supplementary Fig. 5) and temperature-programmed desorption mass spectrometry (TPD-MS) (Supplementary Fig. 6).

**Figure 2:**
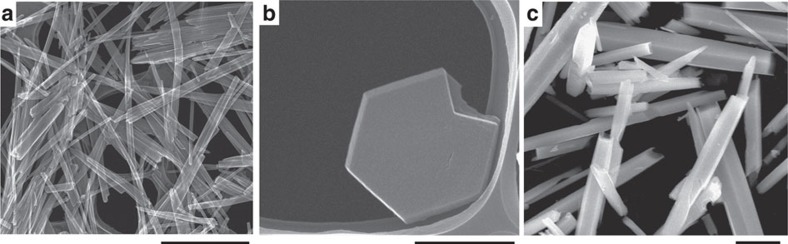
SEM images. (**a**) Mo–Te oxide, scale bar, 3 μm; (**b**) Mo–Se oxide, scale bar, 800 nm; and (**c**) Mo–Te oxide synthesized under refrigeration , scale bar, 5 μm.

Morphologies of the as-synthesized materials were characterized by scanning electron microscopy (SEM). Interestingly, the morphologies of the Mo–Te and Mo–Se oxides were completely different. Mo–Te oxide was a rod-like material (Fig. 2), whereas Mo–Se oxide was a plate-shaped material.

The hydrothermally synthesized Mo–Te and Mo–Se oxides were too small to perform single-crystal analysis (Fig. 2a,b). A large single crystal of Mo–Te oxide was obtained by low-temperature crystallization (see Methods section). The SEM images in Fig. 2a,c show that the Mo–Te oxide synthesized by the low-temperature method was much larger than that synthesized by the hydrothermal method. XRD and FTIR confirmed that the basic structure of the material obtained from the low-temperature synthesis was identical to that of the material obtained from hydrothermal synthesis (Supplementary Fig. 4b).

**Figure 3:**
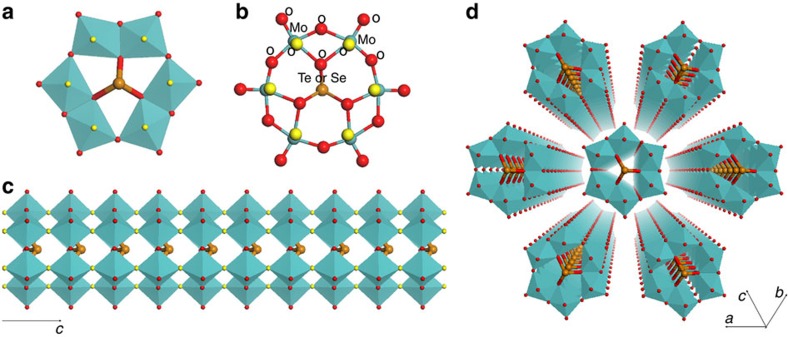
Structural representations. (**a**) Polyhedral representation of a hexagonal unit of [Te^IV^Mo^VI^_6_O_21_]^2−^ or [Se^IV^Mo^VI^_6_O_21_]^2−^. (**b**) Ball-and-stick representation of a hexagonal unit of [Te^IV^Mo^VI^_6_O_21_]^2−^ or [Se^IV^Mo^VI^_6_O_21_]^2−^ with labels. (**c**) A single molecular wire of Mo–Te oxide. The bridge oxygen atoms that connect the hexagonal units are highlighted in yellow. (**d**) Assembly of single molecular wires into crystalline Mo–Te oxide (or Mo–Se oxide). Mo: blue, Te (Se): brown and O: red.

**Figure 4:**
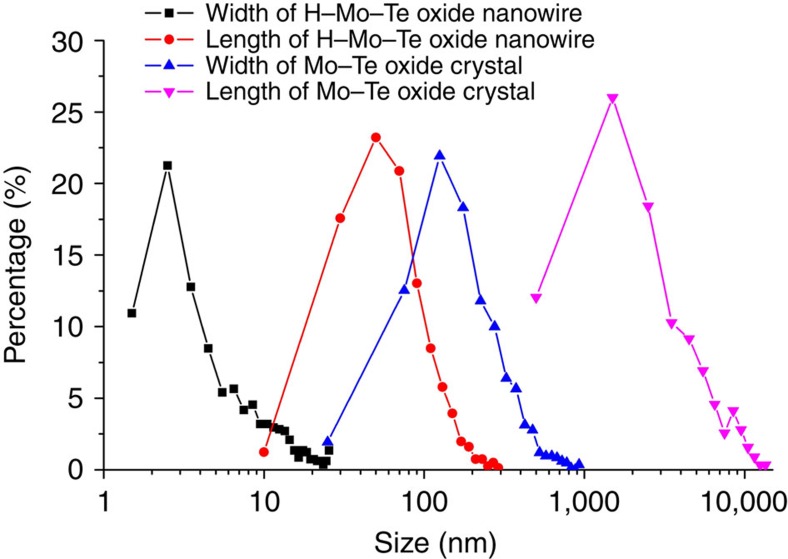
Comparison of morphology and size. Size distributions of the Mo–Te oxide crystals from SEM and the H–Mo–Te oxide molecular wires from TEM. More than 800 particles were counted.

Single-crystal X-ray structural analysis combined with elemental analysis showed that six Mo–O octahedra surrounded one Te ion in the *a*–*b* plane, forming a molecular unit of [Te^IV^Mo^VI^_6_O_21_]^2−^. The Mo–O octahedra units were connected to each other through two edge-sharing oxygen atoms on one side with equatorial Mo–O bond lengths of 1.97(4)–1.99(6) Å and a corner-sharing oxygen atom on another side with equatorial Mo–O bond lengths of 1.79(6)–1.84(5) Å (Fig. 3a,b). The Te ion inside of the Mo–O cluster was coordinated to three oxygen atoms (Te–O bond lengths of 2.05(3)–2.05(4) Å), and a lone pair of Te ions was directed parallel along the *c* direction. The distance between the Te ions was 3.94 Å, and thus there was no interaction between the Te ions. The hexagonal [TeMo_6_O_21_]^2−^ units were stacked along the *c* axis through sharing of the axial oxygen atoms with Mo (axial Mo–O bond lengths of 1.90(5)–2.15(5) Å) to form prismatic clusters (Fig. 3c) as molecular wires. The molecular wires were further packed parallel in a hexagonal manner to form the material (Fig. 3d), and ammonium cations and water were present in between the molecular wires. The nanowires were ca. 1.2 nm in width and micrometre scale in length. The size distribution of the Mo–Te oxide crystals based on SEM images (Fig. 4) showed that most of the particles had widths of 50–200 nm and lengths of 500–3,000 nm with distribution maxima at 150 nm in width and 1,500 nm in length, indicating that over 3,800 hexagonal [TeMo_6_O_21_]^2−^ units accumulated along the *c* direction to form the material. The Mo–Te oxide crystal typically terminated at a sharp tip with a size of 150 × 150 nm in the *a*–*b* plane, with each bundle containing over 14,000 ordered single molecular wires.

The Mo–Se oxide structure was proposed on the basis of powder diffraction Rietveld analysis and elemental analysis (Mo:Se=6:1), showing that the basic structure of the material was the same as that of Mo–Te oxide. The structures of Mo–Te oxide and Mo–Se oxide were refined with Rietveld refinement. The resulting simulated patterns of the materials were similar to those of the experimental data, and the *R*_wp_ values of the Mo–Te and Mo–Se oxides were 7.06% and 7.49%, respectively, indicating that the proposed structures were correct (Fig. 1). The powder samples were pure because no obvious additional peaks were observed.

According to the structural analysis, ultraviolet–vis spectra, and elemental analysis, the chemical formulae were estimated to be (NH_4_)_2_[Te^IV^Mo^VI^_6_O_21_]·3H_2_O for Mo–Te oxide and (NH_4_)_2_[Se^IV^Mo^VI^_6_O_21_]·2H_2_O for Mo–Se oxide.

The molecular wires of Mo–Te oxide and Mo–Se oxide were inorganic polymers formed by accumulation of the [X^IV^Mo^VI^_6_O_21_]^2−^ (X=Te or Se) monomers along the *c* direction; their monomers, with the same or iso-structural molecular structure, have been previously synthesized using bidentate organic ligands, such as carboxylates, to stabilize the monomer structure^29^,^30^.

### Isolation of molecular wire from crystals

**Figure 5:**
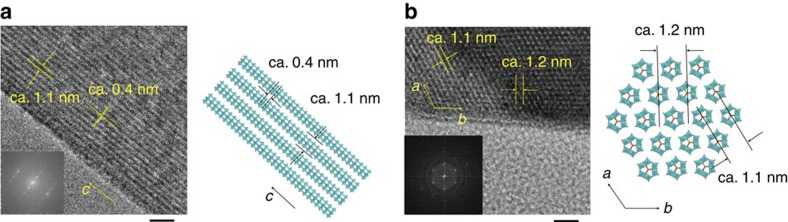
Comparison of polyhedral structural images with HR-TEM images. (**a**) Mo–Te oxide, scale bar, 5 nm and (**b**) Mo–Se oxide, scale bar, 5 nm. Insert images: power spectra.

The order of the molecular wires in the Mo–Te oxide and Mo–Se oxide crystals were observed by high-resolution transmission electron microscopy (HR-TEM) as shown in Fig. 5. Because of the different crystal orientations of Mo–Te oxide and Mo–Se oxide, different lattice planes were observed. In the case of Mo–Te oxide, the molecular wires were packed parallel to the *c* direction with distances of ca. 1.1 nm, and the hexagonal [TeMo_6_O_21_]^2−^ building blocks were accumulated with distances of ca. 0.4 nm, which corresponded to *d* values of (100) and (001) planes, respectively (Fig. 5a). The HR-TEM image of Mo–Se oxide showed a hexagonal arrangement of the molecular wires in the *a*–*b* plane (Fig. 5b). The HR-TEM images were in good agreement with the crystal structures from the structural analysis.

**Figure 6:**
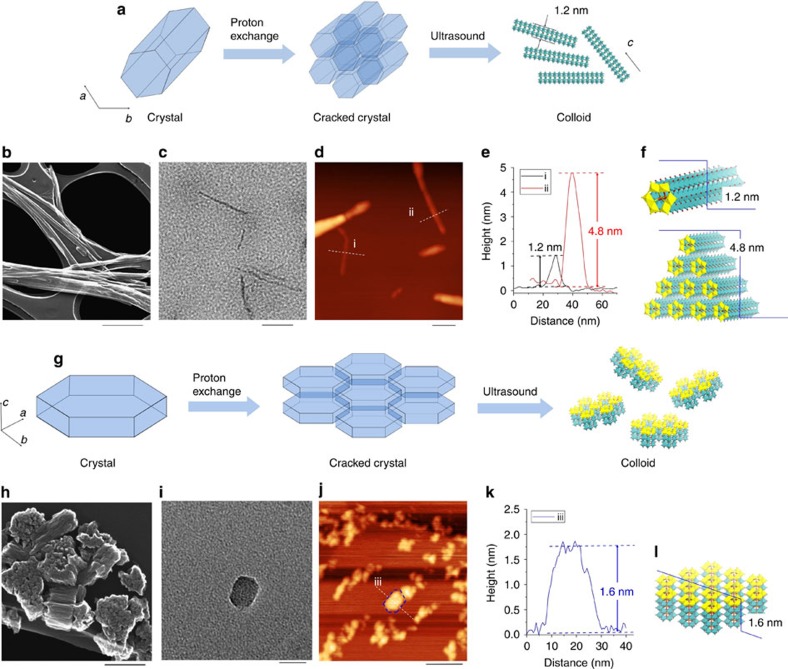
Isolation of crystals into molecular wires. (**a**) Isolation of H–Mo–Te oxide to obtain small particles. (**b**) SEM images of H–Mo–Te oxide, scale bar, 1 μm. (**c**) HR-TEM images of a colloid sample of H–Mo–Te oxide, scale bar, 50 nm. (**d**) AFM images of a colloid sample of H–Mo–Te oxide, scale bar, 50 nm. (**e**) Line profiles from the AFM images. (**f**) Proposed structures from the line profile analysis, with yellow highlighting the *a*–*b* plane of the materials. (**g**) Isolation of H–Mo–Se oxide to obtain small particles. (**h**) SEM images of H–Mo–Se oxide, scale bar, 200 nm. (**i**) HR-TEM images of a colloid sample of H–Mo–Se oxide, scale bar, 20 nm. (**j**) AFM images of a colloid sample of H–Mo–Se oxide, scale bar, 50 nm. (**k**) Line profiles from the AFM images. (**l**) Proposed structures from the line profile analysis, with yellow highlighting the *a*–*b* plane of the materials.

The molecular wire arrays in the crystals were readily disassembled to form single molecular wires by a simple process (Fig. 6a). Ion exchange with protons made the molecular wire isolated from the crystal easily. Ammonium cations in Mo–Te oxide and Mo–Se oxide were replaced by protons to form proton-exchanged materials, denoted as H–Mo–Te oxide and H–Mo–Se oxide, after reaction at room temperature for 15 h. The resulting solids were characterized by powder XRD and FTIR. The diffraction peaks of both materials broadened after proton exchange, indicating that the size of the materials decreased (Supplementary Fig. 4). Furthermore, all characteristic infrared bands of the materials were retained in the materials after proton exchange, demonstrating that the molecular structures of the materials did not change after ion exchange with protons (Supplementary Fig. 4). Elemental analysis confirmed that approximately half of the ammonium cations were replaced by protons, and formulae of the proton-exchanged materials were estimated to be (NH_4_)_1.1_[H_0.9_TeMo_6_O_21_]·5H_2_O and (NH_4_)_1.1_[H_0.9_SeMo_6_O_21_]·6H_2_O.

Proton exchange helped to crack the materials. H–Mo–Te oxide remained a rod-shaped material after proton exchange, and several open gaps could be observed in the crystal surface (Fig. 6b). Crystal breakage after proton exchange was more evident in H–Mo–Se oxide. The material was no longer a plate-like shape compared with the as-synthesized material. The crystals were broken into several small rods (Fig. 6h).

H–Mo–Te oxide could be disassembled into thin rod-like particles and even single molecular wires by dispersion in ethanol with ultrasound. The material was characterized by HR-TEM, which confirmed that nanowires with smaller sizes were generated after ultrasound, and some isolated molecular wires with widths of 1.5 nm (Fig. 6c; Supplementary Fig. 7) were observed. The isolated nanowires were further characterized by atomic force microscopy (AFM). An AFM image of H–Mo–Te oxide after isolation experiments revealed wire-shaped particles. Some ultrathin nanowires were observed in the AFM image, and the thicknesses of typical particles in Fig. 6d were ca. 1.2 nm (particle i) and ca. 4.8 nm (particle ii). The thickness of particle (i) was consistent with that of a single H–Mo–Te oxide molecular wire deduced from the crystallographic data on Mo–Te oxide (Fig. 6e), and particle (ii) was ascribed to a structure with four layers of molecular wires (Fig. 6e). One possible structure is shown in Fig. 6f. The width of the particles appeared to be much larger than that of a single molecular wire (1.2 nm) in the AFM image, which resulted from the large size of the AFM cantilever compared with that of the particles (Supplementary Fig. 8).

The size distribution of the H–Mo–Te oxide nanowires (Fig. 4; Supplementary Fig. 9) indicated that most of the nanowires had widths <10 nm and length between 20 and 80 nm, which were much smaller than those of the Mo–Te oxide crystals.

The H–Mo–Te oxide nanowires were recovered from ethanol by evaporating the ethanol. The recovered material was characterized by XRD (Supplementary Fig. 10). The diffraction peak positions of the recovered H–Mo–Te oxide were similar to those of the as-synthesized Mo–Te oxide, but the peaks of the recovered H–Mo–Te oxide had broadened; this indicated that the basic structure of the wires did not change, but the ordering of the nanowires decreased during the isolation process. These results indicate that the material dispersed in ethanol was a mixture of single molecular wires and nanowires with hexagonally packed molecular wires. The crystallite size estimated by the Scherrer equation using the (100) peak was 16 nm, the order of which was the same as that obtained from the TEM observations.

TEM (Fig. 6i) and AFM (Fig. 6j) images showed that the H–Mo–Se oxide colloid was composed of small nanoplates, which was different from H–Mo–Te oxide. The thickness of the hexagonal-shaped nanoplates was ∼1.6 nm (Fig. 6k), which corresponded to four layers of (NH_4_)_1.1_[H_0.9_SeMo_6_O_21_] along the *c* axis (Fig. 6l).

### Acid catalysis of the H–Mo–Te oxide nanowires

**Figure 7:**
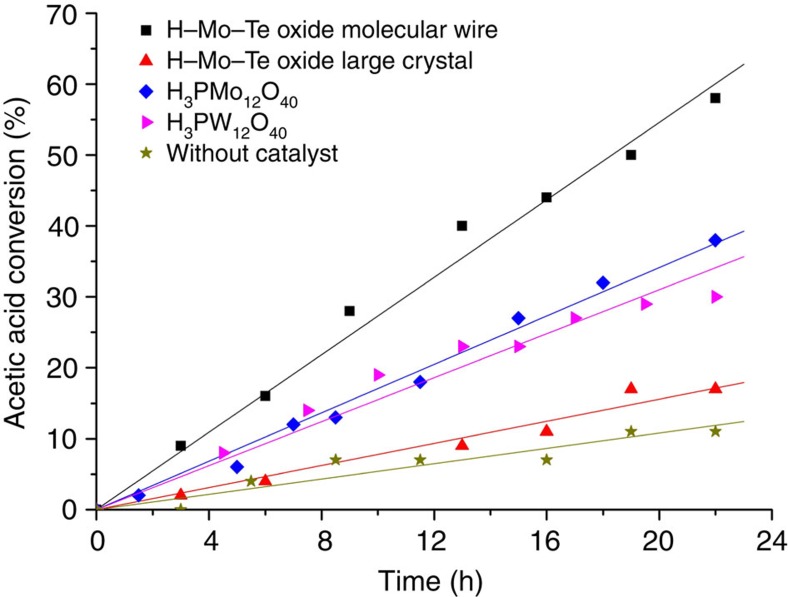
Ethanol esterification with acetic acid. Reaction conditions, catalyst, 0.1 mg; ethanol, 5 ml; acetic acid, 0.1 ml; decane as internal standard, 0.1 ml; reaction temperature, 365 K. The selectivity of ethyl acetate was 99% and the carbon balance was over 95% in all cases.

Nanomaterials are anticipated to have applications in catalysis, because nanomaterials have a large surface area due to their small particle size. Approximately half of the ammonium cations in Mo–Te oxide were able to be replaced by protons, and the material was thus acidic. H–Mo–Te oxide was used to esterify ethanol with acetic acid at 365 K, and the results are summarized in Fig. 7. The acetic acid conversion of large H–Mo–Te oxide particles before ultrasound treatment (17%) was close to that in the reaction without a catalyst (11%), indicating that large H–Mo–Te oxide particles before ultrasound treatment had low catalytic activity under the present conditions. The catalytic activity of the H–Mo–Te oxide nanowires after ultrasound treatment (58%) was much higher than that of the large H–Mo–Te oxide particles due to the smaller particle size of the former, as well as the catalytic activities of other homogeneous acid catalysts such as H_3_PW_12_O_40_ (30%) and H_3_PMo_12_O_40_ (38%). The catalyst was recyclable and reused three times with only a slight decrease in activity (Supplementary Fig. 11).

### Altering the band gap of Mo–Te oxide

1D nanostructured materials have potential applications in the emerging field of nanoelectronics, including applications as functional components and as conductive connections^31^,^32^,^33^. Nanostructured molybdenum oxides have been reported as good candidates for applications in electronic devices.^28^ As a molybdenum oxide-based molecular wire, Mo–Te oxide is also expected to have applications in electronic devices. Electroconductivity of a material is important for electronic applications. However, bulk molybdenum (VI)-based oxides, such as α-MoO_3_, were shown to have poor electroconductivity because of the large band gap of the material (>2.7 eV)^34^, and the material was therefore not suitable as a semiconductor^28^. Reducing the Mo^VI^ ions by reaction under reducing conditions decreased the band gap of the material^28^,^33^.

For Mo–Te oxide, the use of heat treatment without an additional reducing agent enables easy manipulation of the oxidation states of the Mo ions in the material, and the band gap of the material can thus be tuned continuously. Mo–Te oxide was calcined under N_2_ (180 ml min^−1^, 1 h, 473–573 K). Powder XRD patterns and FTIR spectra of the calcined samples were similar to those of the as-synthesized material, indicating that the basic structure did not change (Supplementary Fig. 12). Further heating of the material damaged its structure (Supplementary Fig. 12g,h). The diffraction peaks shifted to a higher angle after the material had been treated at a high temperature, and this shift in diffraction peaks was caused by a decrease in the distances between the molecular wires after removal of some water or ammonia from the material. The calcined Mo–Te oxide at 573 K was further characterized, and it remained a rod-shaped material (Supplementary Fig. 13a). The particle size and morphology did not change with calcination, which was indicated by the size distribution (Supplementary Fig. 14). The arrangement of the molecular wire was observed by TEM (Supplementary Fig. 13b) and was in good agreement with the powder XRD patterns. Thus, calcination did not affect the morphology of the material.

**Figure 8:**
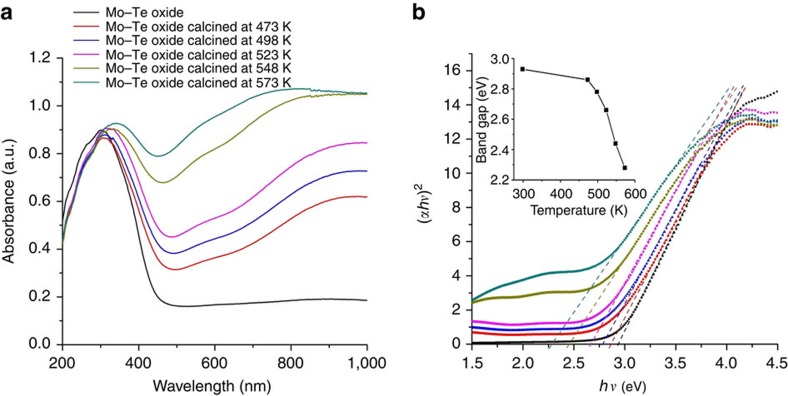
Band gap change in Mo–Te oxide. (**a**) Diffuse reflectance ultraviolet–vis spectra of the materials and (**b**) plots of *hν* versus (α*hν*)^2^, where *α* stands for the absorption coefficient: as-synthesized Mo–Te oxide (black) and Mo–Te oxide calcined for 1 h at 473 K (red), 498 K (blue), 523 K (pink), 548 K (yellow) and 573 K (cyan). Insert: band gap changed with the treatment temperature; dotted lines indicate the band gaps.

Photo images of the Mo–Te oxide showed that the colour of the material gradually changed to blue with an increase in the treatment temperature (Supplementary Fig. 15), indicating that reduced Mo species were generated in the material. The resulting materials were further characterized by ultraviolet–vis spectroscopy, which revealed that Mo ions in the material were reduced by heating (Fig. 8a). The band gap calculated from the ultraviolet–vis spectra indicated that the band gap had decreased with the reduction of Mo ions in the material (Fig. 8b). The band gap of the original Mo–Te oxide was 2.93 eV. After calcination, the band gap of the material decreased to 2.28 eV. The oxidation states of the Mo and Te ions in the material were further studied by XPS. Most of the Mo^VI^ ions were reduced to Mo^V^ ions after heat treatment at 573 K under N_2_, whereas the Te ions in the material were not oxidized (Supplementary Fig. 1f–h). TPD-MS profiles (Supplementary Fig. 6a) of Mo–Te oxide revealed that N_2_ had desorbed from the material during heat treatment, indicating that the NH_3_ in the material had been oxidized to N_2_, whereas the Mo^VI^ ions had been reduced to Mo^V^ ions. Therefore, the Mo ions were reduced in their reaction with NH_3_ in the material. It has been reported that any charge transfer or *n*-type electron doping in a conduction band causes lattice distortion of Mo-based oxides and consequently a reduction in the band gap^35^. Regarding Mo–Te oxide under heat treatment, electrons were transferred from NH_4_^+^ to the conduction band of the material (formed in the molecular wires from the *d*-orbitals of Mo), and therefore the band gap could be reduced. The XRD peak at ca. 22 degree shifted after calcination (Supplementary Fig. 12C), which was ascribed to lattice distortion of the molecular wire.

## Discussion

It is interesting that Mo–Te oxide and Mo–Se oxide had similar basic structure, but different morphologies. The lattice parameters of Mo–Te oxide and Mo–Se oxide were similar, and the negative charge and counter cation were the same. Mo–Te oxide was a rod-like material that exhibited preferential crystal growth along the *c* direction. By contrast, Mo–Se oxide was a hexagonal plate-shaped material that preferred crystal growth along the *a*–*b* plane. Several factors affecting this difference in morphology were considered.

First, the difference in the central elements, Te^IV^ and Se^IV^, was considered. In *α*-Keggin-type POMs, [X^*n*+^W_12_O_40_]^(8−*n*)−^ (X:Si(*n*=4), Ge(*n*=4), P (*n*=5), As (*n*=5), Co(*n*=2) and Zn(*n*=2)), properties such as the redox potential and affinity for protons depend on the identity of the central element. These differences result from the ionic size and electronegativity of the central element^36^. Differences in the ionic radii and electronegativity of Te^IV^ and Se^IV^ might have affected the morphology of the Mo–Te oxide and Mo–Se oxide crystals.

Second, the synthetic preparations of the materials were different. Soluble SeO_2_ was used as a Se^IV^ source in Mo–Se oxide, whereas Te(OH)_6_ with a reducing reagent (VOSO_4_) was used as a Te^IV^ source in Mo–Te oxide. An obvious difference is that a large amount of Mo–Se oxide (ca. 10% of the yield) was formed within 10 min at room temperature (material characterization in Supplementary Fig. 16 and Supplementary Methods), whereas nearly no solid was observed in the Mo–Te oxide system even after 1 day. Only a small amount of Mo–Te oxide (ca. <0.06% of the yield) was produced after 2 days at room temperature (Supplementary Fig. 17; Supplementary Methods). This indicates that formation rate of Mo–Se oxide was faster than that of Mo–Te oxide. Mo–Se oxide was produced immediately after mixing the Mo and Se sources, which might result from the high concentration of Se^IV^ species in the initial solution due to the soluble SeO_2_. In Mo–Te oxide, Te^IV^ was produced slowly *in situ* by reduction of Te(OH)_6_ by VOSO_4_ (Supplementary Fig. 18). Furthermore, Te^IV^ is typically insoluble in water compared with Se^IV^, which might affect the concentration of Te^IV^ (Supplementary Fig. 19). These factors could result in the slow generation of Mo–Te oxide. Therefore, the different synthetic conditions and formation processes might also affect the morphology of the materials.

Mo–Se oxide can also be synthesized through reduction of Se(OH)_6_ with VOSO_4_ (synthesis and characterization in Supplementary Methods, Supplementary Fig. 20 and Supplementary Fig. 21), which slowly generated Se^IV^ (Supplementary Fig. 18). FTIR (Supplementary Fig. 20b) indicated that the structures of the molecular wires in the resulting material were identical to that obtained using SeO_2_. Powder XRD Rietveld analysis indicated an identical molecular structure in the molecular wires with slightly different packing (Supplementary Fig. 20; Supplementary Fig. 21). SEM images and size distributions (Supplementary Fig. 22) showed that the *a*–*b* plane of the material using Se(OH)_6_ was much smaller than that using SeO_2_. This result also supports the fact that synthetic conditions affected the morphology of the products.

Proton exchange facilitates the isolation of the molecular wires from the Mo–Te oxide crystal. The density functional theory (DFT) calculation was used to further understand the process. In the Mo–Te oxide crystals, there were three main interactions organizing the molecular wires into the crystal: electrostatic interactions between anionic molecular wires and ammonium cations, hydrogen bond interactions and van der Waals forces. Generally, the electrostatic interaction is stronger than the other two interactions. Weakening the electrostatic interaction through proton exchange drove isolation from the crystal to form the molecular wires, as demonstrated by the DFT calculation.

**Figure 9:**
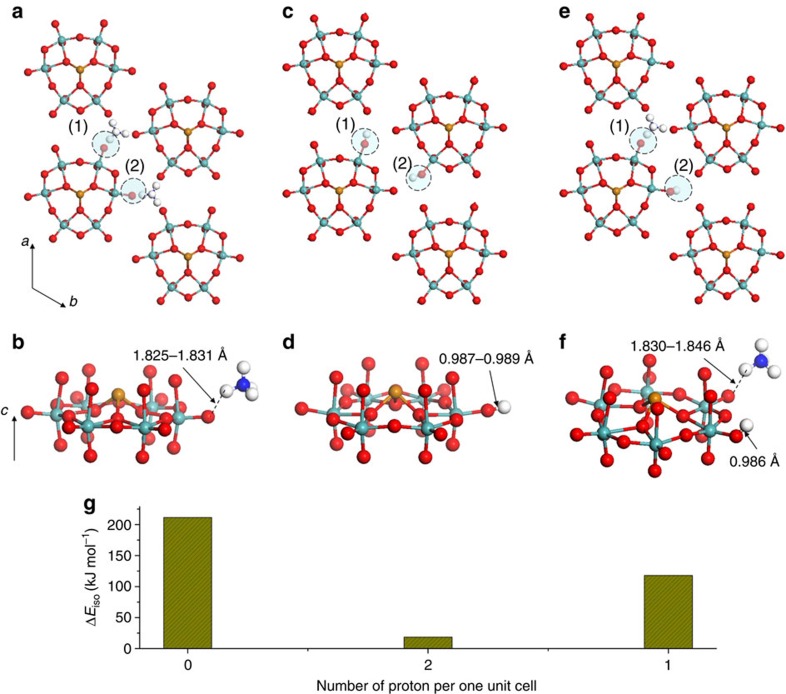
Structure in unit cells after geometry optimization. (**a**) Mo–Te oxide with two ammonium cations in a unit cell. (**b**) Magnified image of a hexagonal unit of [TeMo_6_O_21_] with an ammonium cation. (**c**) Mo–Te oxide with two protons in a unit cell. (**d**) Magnified image of a hexagonal unit of [TeMo_6_O_21_] with a proton. (**e**) Mo–Te oxide with a ammonium cation and a proton in a unit cell. (**f**) Magnified image of a hexagonal unit of [TeMo_6_O_21_] with an ammonium cation and a proton. (**g**) Calculated Δ*E*_iso_.

Single-crystal structure analysis revealed that the distance of the N in NH_4_^+^ and the O in the Mo=O terminal bond (O(Mo=O_t_)) was ca. 2.669–2.814 Å, indicating the presence of electrostatic interactions in the materials. After optimizing the geometry with the DFT calculation, the distance of the N in NH_4_^+^ and O(Mo=O_t_) was 2.753–2.762 Å, similar to the single-crystal data. Moreover, the DFT calculation demonstrated that the distance between the H in NH_4_^+^ (H(NH_4_^+^)) and O(Mo=O_t_) was 1.825–1.831 Å (Fig. 9a,b), indicating no covalent bond between H(NH_4_^+^) and O(Mo=O_t_). Therefore, electrostatic interactions existed in the material. The chemical formula of molecular wire was (NH_4_)_2*n*_[TeMo_6_O_21_]_*n*_, indicating that [TeMo_6_O_21_]^2−^ interacted with two NH_4_^+^.

In the second model, all ammonium cations were ideally replaced by protons. The DFT calculation showed that two Mo=O_t_ bonds were protonated because the O–H bond length was 0.987–0.989 Å (Fig. 9c,d). Protonation of the terminal M=O is common in POMs. Therefore, after proton exchange, the anionic molecular wire was neutralized by protons, destroying the electrostatic interactions.

The third model is the closest to the proton-exchanged material (Fig. 9e,f), in which half of the ammonium cations were replaced by protons. After geometry optimization only one Mo=O_t_ was protonated in a cell. The anionic molecular wire was partly neutralized by proton exchange. The chemical formula of the molecular wire was (NH_4_)_*n*_[HTeMo_6_O_21_]_*n*_, indicating that one [HTeMo_6_O_21_]^−^ interacted with only one NH_4_^+^. Therefore, the electrostatic interaction was weakened by proton exchange.

The energy to isolate of the crystal and form the molecular wire (Δ*E*_iso_), denoted as Δ*E*_iso_=*E*_molecular wire_−*E*_crystal_, was calculated; Δ*E*_iso_ decreased with increasing protons in the material (Fig. 9g). Thus, proton exchange decreased the electrostatic interaction (Δ*E*_iso_), which caused the crystal to crack into small molecular wires.

Furthermore, Raman spectra and FTIR spectra (Supplementary Fig. 23) contained shifts in the Raman band at 964 cm^−1^ and the IR band at 939 cm^−1^ after proton exchange, indicating that the Mo=O bond lengths were altered after proton exchange and protonation of Mo=O^37^.

In summary, self-assembly of Mo–O octahedra with Se^IV^ or Te^IV^ formed the transition metal oxide-based molecular wires. The structures of Mo–Te oxide and Mo–Se oxide were determined by single-crystal X-ray analysis and powder XRD Rietveld analysis combined with XPS, FTIR and elemental analysis. The materials were composed of [X^IV^Mo^VI^_6_O_21_]_*n*_ (X=Se and Te) hexagonal-shaped 1D molecular wires with water and NH_4_^+^. Because of their unique structural properties, these materials will give rise to new transition metal oxide nanowire technologies based on their 1D properties.

The metal oxide molecular wires exhibited acid-catalyst activities, which will open up a new field of heterogeneous catalysts based on molecular wires. Moreover, the band gap of the material can be easily manipulated, which may alter the electroconductivity of the material. These molecular wire-based materials are expected to have applications in the fields of thermochromic materials and semiconductors, as well as other related fields.

## Methods

### Synthesis of Mo–Te oxide

(NH_4_)_6_Mo_7_O_24_·4H_2_O (1.766 g, 10 mmol based on Mo) was dissolved in 20 ml of water, followed by addition of Te(OH)_6_ (0.391 g, 1.7 mmol) to the (NH_4_)_6_Mo_7_O_24_·4H_2_O solution to form solution A. Then, VOSO_4_·5H_2_O (0.6438, g, 2.5 mmol) was dissolved in 20 ml of water to form solution B. Solution B was poured rapidly into solution A. The mixture was stirred at room temperature for 10 min and degassed by N_2_ bubbling for 10 min (pH 2.8). The mixture was introduced into the 50-ml Teflon-liner of a stainless-steel autoclave, which was heated at 448 K for 24 h. After the autoclave had cooled to room temperature, the resulting solid was recovered from the solution by filtration. The obtained solid was washed with 10 ml of water three times and dried at 353 K overnight. Then, 1.07 g of Mo–Te oxide was obtained (yield of 58% based on Mo). Elemental analysis: calculated for N_2_Mo_6_Te_1_O_24_H_14_: N, 2.48; Mo, 50.98; Te, 11.30; H, 1.24; V, 0, found: N, 2.49; Mo, 51.59; Te, 11.13; H, 1.22; V, 0.

### Synthesis of large Mo–Te oxide crystals

(NH_4_)_6_Mo_7_O_24_·4H_2_O (1.766 g, 10 mmol based on Mo) was dissolved in 20 ml of water, followed by addition of Te(OH)_6_ (0.391 g, 1.7 mmol) to the (NH_4_)_6_Mo_7_O_24_·4H_2_O solution to form solution A. Then, VOSO_4_·5H_2_O (0.6438, g, 2.54 mmol) was dissolved in 20 ml of water to form solution B. Solution B was poured rapidly into solution A. The mixture was stirred at room temperature for 10 min and degassed by N_2_ bubbling for 10 min (pH of 2.8). The solution was sealed and stored in a refrigerator for ∼3 months. The large well-crystallized Mo–Te oxide was collected by centrifugation (3,500 r.p.m., 5 min). Then, 5 mg of Mo–Te oxide was obtained (yield of 0.3% based on Mo).

### Synthesis of Mo–Se oxide

(NH_4_)_6_Mo_7_O_24_·4H_2_O (1.766 g, 10 mmol based on Mo) was dissolved in 20 ml of water, followed by addition of SeO_2_ (0.189 g, 1.7 mmol) to the (NH_4_)_6_Mo_7_O_24_·4H_2_O solution. The pH of the solution was adjusted to 2.8 by H_2_SO_4_ (1 M), and the solution was stirred at room temperature for 10 min (yellow solid was generated during this process) and degassed by N_2_ bubbling for 10 min. The mixture was introduced into the 50-ml Teflon-liner of a stainless-steel autoclave, which was heated at 448 K for 24 h. After the autoclave had cooled to room temperature, the resulting solid was recovered from the solution by filtration. The obtained solid was washed with 10 ml of water three times and dried at room temperature overnight. Then, 0.17 g of Mo–Se oxide was obtained (yield of 9.6%). Elemental analysis: calculated for N_2_Mo_6_Se_1_O_23_H_12_: N, 2.64; Mo, 54.17; Se, 7.43; and H, 1.13, found: N, 2.72; Mo, 54.19; Se, 7.58; and H, 1.25.

### Ion exchange with protons

Mo–Te oxide or Mo–Se oxide (0.6 g) was dispersed in 24 ml of water. HCl (36%, 2 ml) was added to the solution. The mixture was stirred for 15 h. The resulting solid was recovered by filtration and dried at room temperature. Elemental analysis: calculated for N_1.1_Mo_6_Te_1_O_26_H_15.3_ (denoted as H–Mo–Te oxide): N, 1.34; Mo, 50.06; Te, 11.10; and H, 1.33, found: N, 1.44; Mo, 50.34; Te, 11.18; and H, 1.51. Elemental analysis: calcualted for N_1.1_Mo_6_Se_1_O_27_H_17.3_ (denoted as H–Mo–Se oxide): N, 1.38; Mo, 51.43; Se, 7.05; and H, 1.55, found: N, 1.52; Mo, 51.68; Se, 7.05; and H, 1.58.

### Separation of molecular wires

H–Mo–Te oxide or H–Mo–Se oxide (5 mg) was dispersed in 5 ml of ethanol, followed by ultrasonication (200 W, 37 kHz) for 1 h. The solution was centrifuged for 2 h (3,500 r.p.m.). The upper 50% of the colloid was used in further characterization. Approximately 2 wt% of H–Mo–Te oxide formed nanoparticles, as determined by measuring the weight of remaining solid in the solution.

### Catalytic activity

A catalyst (0.1 g) was dispersed in 5 ml of ethanol. Acetic acid (0.1 ml) and decane (0.1 ml) were added to the ethanol solution. The mixture was heated at 365 K. The reaction was monitored by gas chromatography with flame ionization detector. Conversion was calculated based on acetic acid. The selectivity was calculated by *n*(ethyl acetate)/*n*(all products), and the carbon balance was calculated by *n*(all products)/*n*(acetic acid), where *n* is the molar amount. The carbon balance was over 95% and the selectivity of ethyl acetate was 99% in all cases.

### Calcination

Mo–Te oxide (0.1 g) was calcined in a glass tube that was set into a furnace under N_2_ flow (180 ml min^−1^) at 473, 498, 523, 548 and 573 K for 1 h with a rate of increase in temperature of 10 K min^−1^.

### Characterization

XRD patterns were obtained on RINT2200 (Rigaku, Japan) with Cu Kα radiation (tube voltage: 40 kV, tube current: 20 and 40 mA for structural analysis). FTIR spectroscopy was carried out on a Perkin Elmer PARAGON 1000 (Perkin Elmer, USA). Diffuse reflectance ultraviolet–vis spectra were obtained using a JASCO V-570 spectrophotometer equipped with an ISN-470 reflectance spectroscopy accessory (JASCO, Japan). The band gap of the material was obtained from the figure of *hν* versus (*αhν*)^2^ via the equation of (*αhν*)^2^=*k*(*hν*−*E*_g_), where *E*_g_ is the band gap; *α* is the absorption coefficient; *hν* is the incident light energy; and *k* is a constant^38^. XPS was performed on a JPS-9010MC (JEOL, Japan). The spectrometer energies were calibrated using the Au 4f_7/2_ peak at 84 eV. Raman spectra were recorded on a Renishaw inVia Raman Microscope (Renishaw, UK).

TPD-MS measurements were carried out from 313 to 893 K at a heating rate of 10 K min^−1^ under He flow (flow rate: 50 ml min^−1^). Samples were set up between two layers of quartz wool. A TPD apparatus (BEL Japan, Inc., Japan) equipped with a quadrupole mass spectrometer (M-100QA. Anelva) was used to detect NH_3_ (*m*/*z*=16), H_2_O (*m*/*z*=18), N_2_ (*m*/*z*=28) and O_2_ (*m*/*z*=32). TG-DTA was carried out up to 773 K at a heating rate of 10 K min^−1^ under nitrogen flow (flow rate: 50 ml min^−1^) on a Thermo plus TG-8120 (Rigaku, Japan).

SEM images were obtained with a HD-2000 (HITACHI) and a JSM-6360LA (JEOL, Japan). TEM images were taken with a 200-kV TEM (JEOL, JEM-2010F, Japan). AFM images were obtained on an Agilent 5500 scanning probe microscope (Agilent Technologies, USA) in air by a silicon cantilever with a 7-nm radius.

Elemental compositions were determined using an inductively coupled plasma atomic emission spectroscopy method (ICPE-9000, Shimadzu, Japan). The CHN elemental compositions were determined in the Instrumental Analysis Division, Equipment Management Center, Creative Research Institution, Hokkaido University.

### Single-crystal X-ray analysis of Mo–Te oxide

The crystals obtained at room temperature were still too small for the diffractometer in the laboratory single-crystal analysis system, and data collection was performed on a high-precision diffractometer installed in the SPring-8 BL40XU beamline^39^,^40^. The synchrotron radiation emitted from a helical undulator was monochromated by an Si(111) channel cut monochromator and focused with a Fresnel zone plate. A Rigaku Saturn724 charge-coupled device detector was used. The measurement was performed at 100 (2) K. An empirical absorption correction based on Fourier series approximation was applied. The data were corrected for Lorentz and polarization effects. The structure was solved by direct methods and refined by full-matrix least-squares (SHELX-97)^41^, where the unweighted and weighted agreement factors of *R*=Σ||*F*_o_|−|*F*_c_||/Σ|*F*_o_| (*I*>2.00*σ*(*I*)) and w*R*=[Σw(*F*o^2^−*F*c^2^)^2^/Σw(*F*o^2^)^2^]^1/2^ were used. Crystallographic data, atom position and bond lengths for Mo–Te oxide are shown in Supplementary Tables 2–4. The Cif file is available in Supplementary Data 1.

### Structure determination by powder X-ray diffraction

The structure of Mo–Se oxide was determined from powder XRD. A powder XRD pattern for structural analysis was obtained on a RINT2200 (Rigaku, Japan) with Cu Kα radiation (tube voltage: 40 kV, tube current: 40 mA, scan speed: 1° min^−1^, step: 0.01°). First, the powder XRD pattern was indexed by the DICVOL06 (ref. 42) and X-cell programs^43^. After performing Pawley refinement, the most reasonable space group was obtained. Then, the Le Bail method^44^ was applied for intensity extraction with the EdPCR program. The initial structure was solved by a charge-flipping algorithm^45^. The positions and types of atoms were obtained by analysing the generated electron density maps (Supplementary Table 5). The initial structure was refined by Rietveld analysis.

### Rietveld refinement

The structures of Mo–Te oxide (from single-crystal analysis) and Mo–Se oxide (from powder XRD) were refined by powder XRD Rietveld refinement^46^. Pattern parameters and lattice parameters of the materials were refined by the Pawley method. The atomic temperature factor of Mo–Te oxide was from the single-crystal analysis. Then, isotropical temperature factors were given for every atom in the initial structure of Mo–Se oxide. Rietveld analysis was initiated with the initial models of the materials, and lattice parameters and pattern parameters were from the Pawley refinement. Every atom position was refined. Occupancy of atoms in the framework was fixed without further refinement, and occupancies of atoms in water and the cations were refined with consideration of elemental analysis results. Finally, the pattern parameters were refined again to obtain the lowest *R*_wp_ value. The crystallographic parameters, atom positions and bond lengths of Mo–Se oxide are shown in Supplementary Table 2, Supplementary Table 6 and Supplementary Table 7, and the Rietveld analysis parameters of Mo–Te oxide and Mo–Se oxide are shown in Supplementary Table 1.

### DFT calculations

The structures of the Mo–Te oxide crystals and the Mo–Te oxide molecular wires were optimized using the DMol^3^ program^47^,^48^. The Perdew–Burke–Ernzerhof generalized gradient functional and DND basis sets were employed. The lattice parameters and atom coordination of the initial structure of the Mo–Te oxide crystal were from the single-crystal analysis. For the Mo–Te oxide molecular wires, the structure was optimized in a cell with lattice parameters of *a*=*b*=30 Å, *c*=3.944 Å and *α*=*β*=*γ*=90°, which insured that the interaction between each molecular wire was negligible. The relative positions of the atoms in the molecular wires were the same as that from the single-crystal analysis.

## Additional information

**Accession codes:** The X-ray crystallographic coordinates for structures reported in this Article have been deposited at the Cambridge Crystallographic Data Centre (CCDC), under deposition number CCDC 1401200. These data can be obtained free of charge from The Cambridge Crystallographic Data Centre via www.ccdc.cam.ac.uk/data_request/cif.

**How to cite this article:** Zhang, Z. *et al.* Ultrathin inorganic molecular nanowire based on polyoxometalates. *Nat. Commun.* 6:7731 doi: 10.1038/ncomms8731 (2015).

## Supplementary Material

Supplementary Figures, Supplementary Tables and Supplementary Methods Supplementary Figures 1-23, Supplementary Tables 1-7 and Supplementary Methods

Supplementary Data 1 Crystallographic Information File for Mo-Te oxide
