# Supplementary material for: Ultrathin inorganic molecular nanowire based on polyoxometalates
Source: Nat Commun. 2015 Jul 3;6:7731. doi: 10.1038/ncomms8731 (PMC4506542; doi:10.1038/ncomms8731)
Supplement: Supplementary Figures, Supplementary Tables and Supplementary Methods — Supplementary Figures 1-23, Supplementary Tables 1-7 and Supplementary Methods [file ncomms8731-s1.pdf]

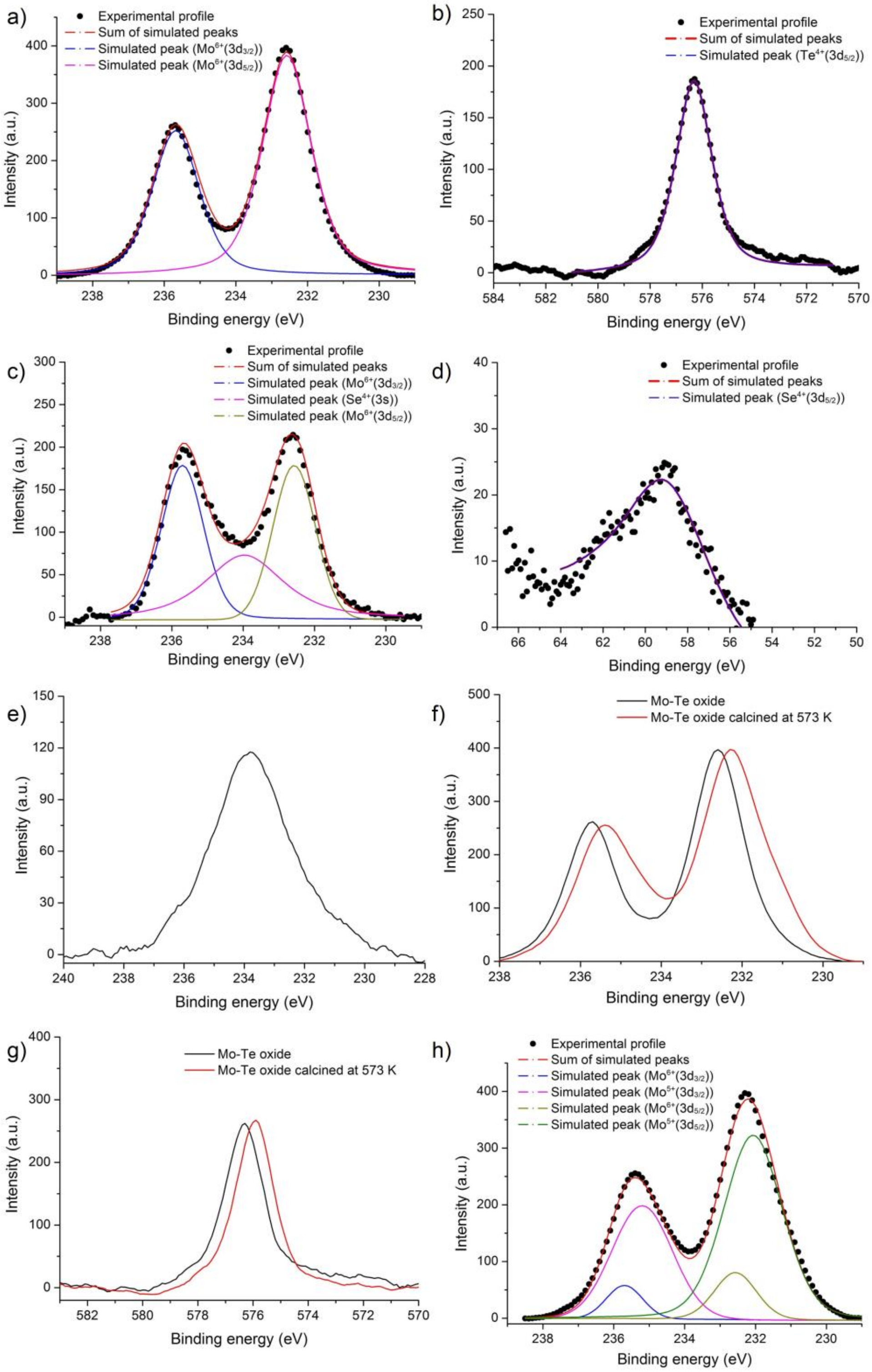

Supplementary Figure 1. XPS spectra and curve fitting results. a) Mo of Mo–Te oxide, b) Te of Mo–Te oxide, c) Mo of Mo–Se oxide (Mo XPS profile was well fitted using $Mo^{VI}(3d_{5/2})$, $Mo^{VI}(3d_{7/2})$, and $Se^{IV}(3s)$, because $Se^{IV}(3s)$ has a signal at 240~230 eV (Supplementary Figure 1e).), d) Se ($3d_{5/2}$) of Mo–Se oxide, e) Se (3s) of SeO_2. XPS spectra comparison of f) Mo and g) Te of Mo–Te oxide (black) and Mo–Te oxide after calcination at 573 K under N_2 flow (red), and h) XPS spectra and curve fitting results of Mo–Te oxide (Mo) calcined at 573 K under N_2 flow.

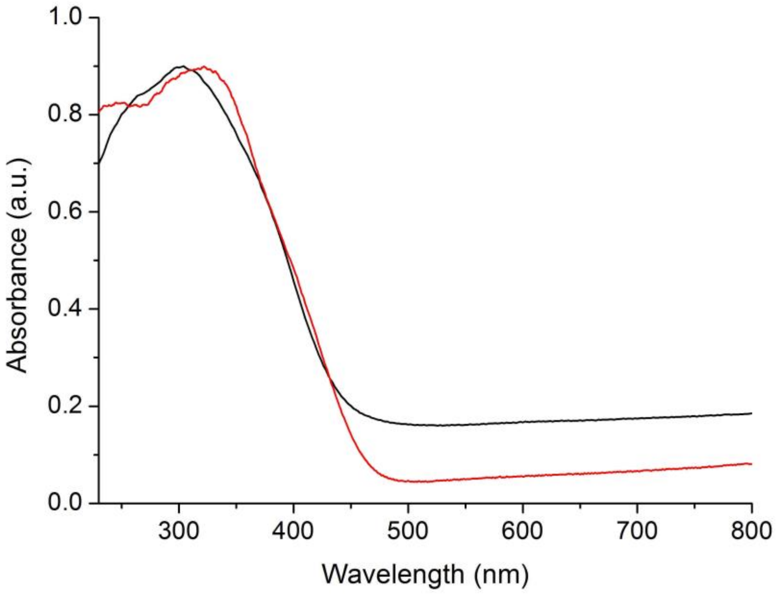

Supplementary Figure 2. DR-UV-vis spectra. Mo–Te oxide (black) and Mo–Se oxide (red).

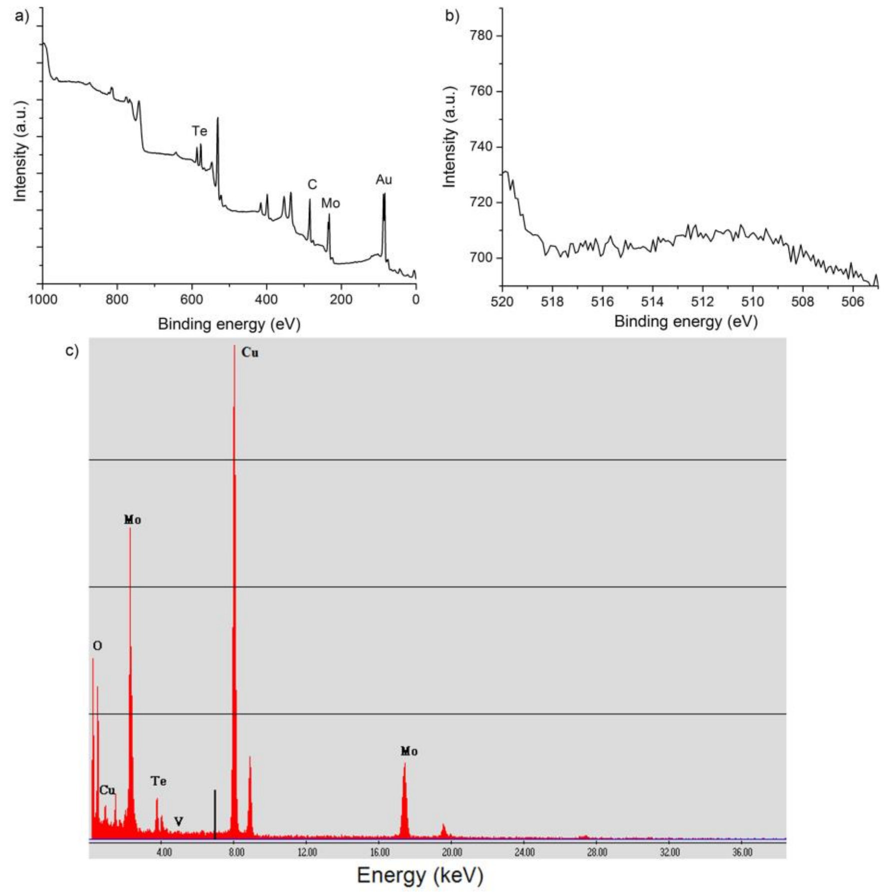

Supplementary Figure 3. XPS and EDX analysis. a) XPS profile at full scale, b) XPS profile in 520~505 eV for V($2p_{3/2}$), and c) EDX of Mo–Te oxide

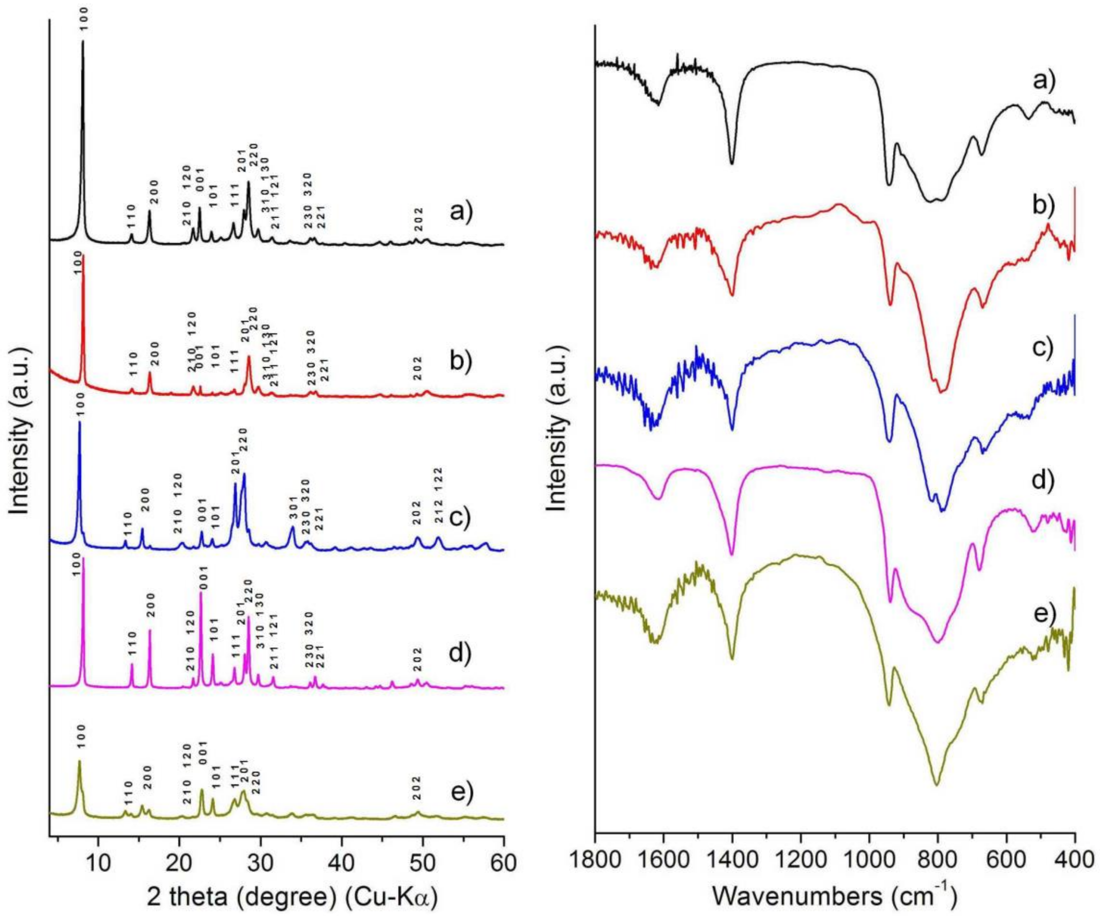

Supplementary Figure 4. XRD patterns (left) and FT-IR spectra (right). a) Mo–Te oxide, b) Mo–Te oxide synthesized under refrigeration, c) H–Mo–Te oxide, d) Mo–Se oxide, and e) H–Mo–Se oxide.

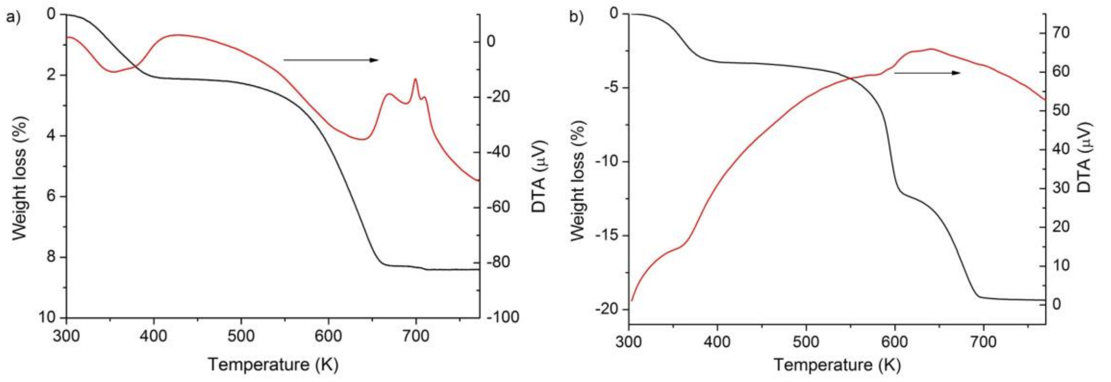

Supplementary Figure 5. **TG-DTA results.** a) Mo–Te oxide and b) Mo–Se oxide under N_2 flow (50 mL min^{-1}).

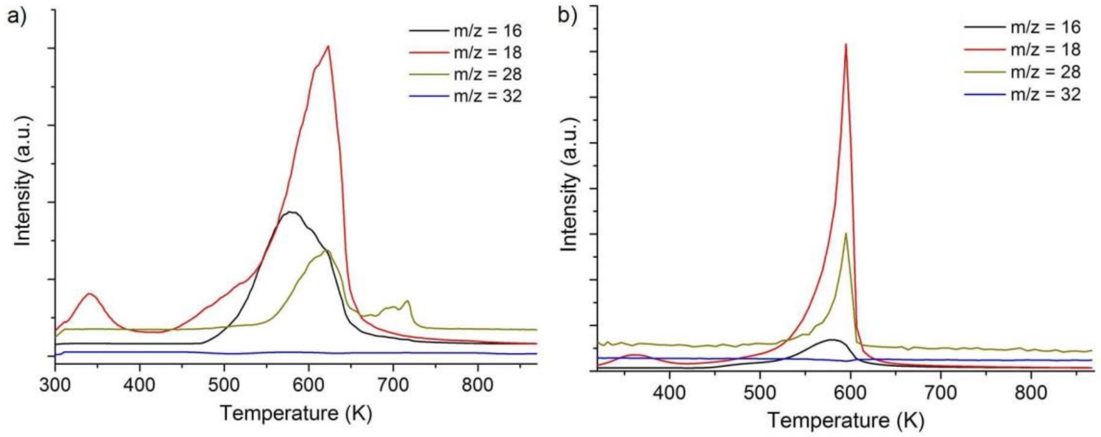

Supplementary Figure 6. TPD-MS profiles. a) Mo–Te oxide and b) Mo–Se oxide. m/*z* = 16, 18, 28, and 32 represent NH_3, H_2O, N_2, and O_2, respectively.

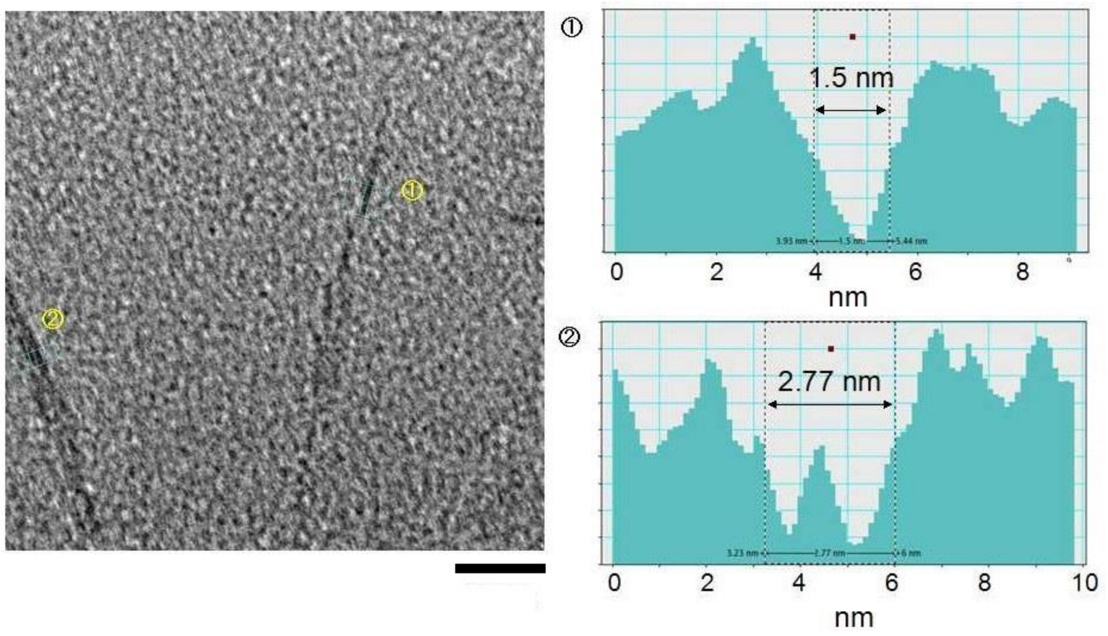

Supplementary Figure 7. TEM image of H–Mo–Te oxide nanowires and corresponding intensity profiles across the oxide nanowires. Separation of the (①) single and (②) double isolated molecular wires is clearly seen in the intensity profiles; scale bar = 20 nm.

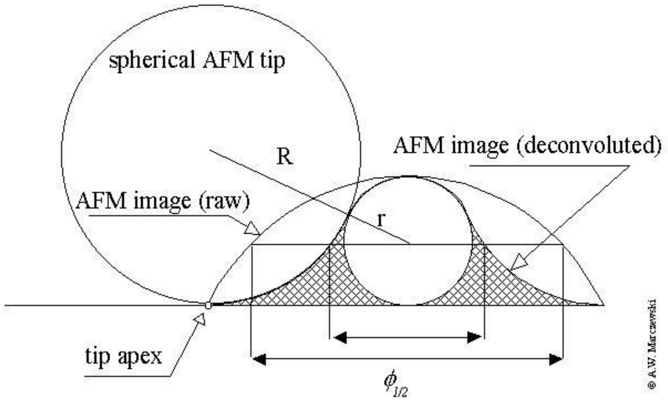

Supplementary Figure 8. Schematic figure for the AFM tips and particles under observation. R (radius of tip) = 7 nm, r (radius of particle) = 1 nm.

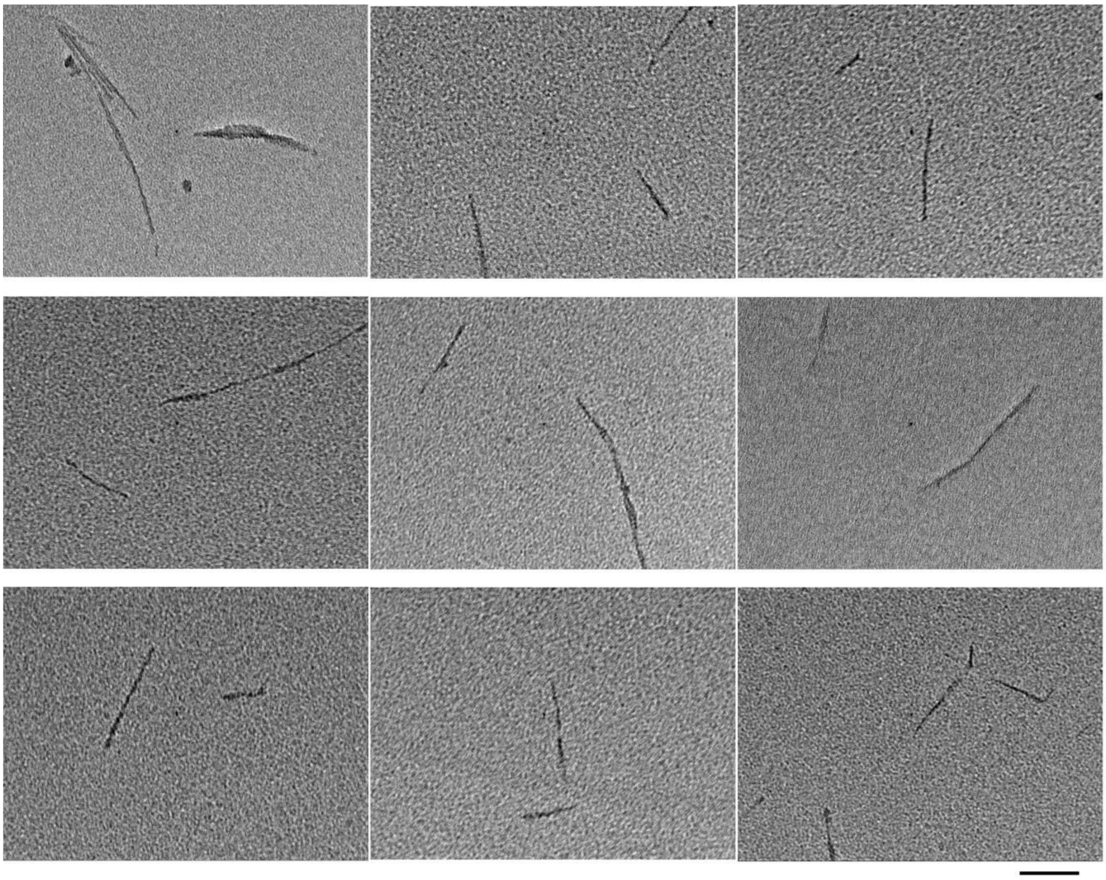

Supplementary Figure 9. Typical TEM images of H–Mo–Te oxide molecular wires. Scale bar = 50 nm.

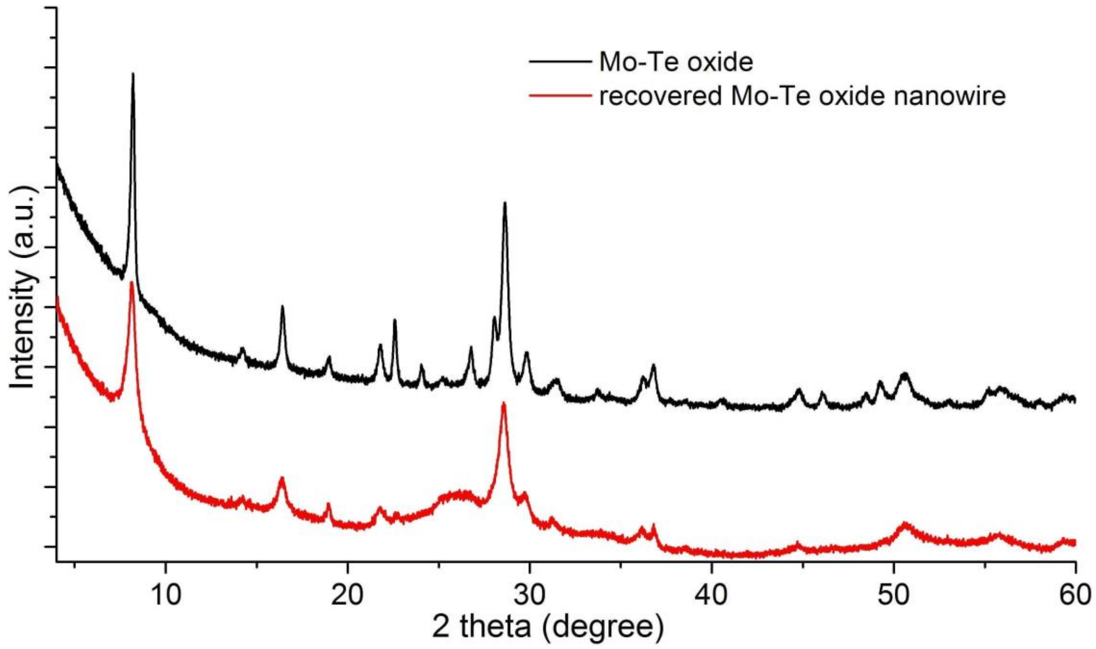

Supplementary Figure 10. XRD patterns of as-synthesized Mo–Te oxide crystals and recovered H–Mo–Te oxide nanowires. Because the amount of recovered Mo–Te oxide nanowires was low, only 1 mg of samples in both cases was used for XRD experiments. The high baseline, especially at a low angle, was due to the small amount of sample.

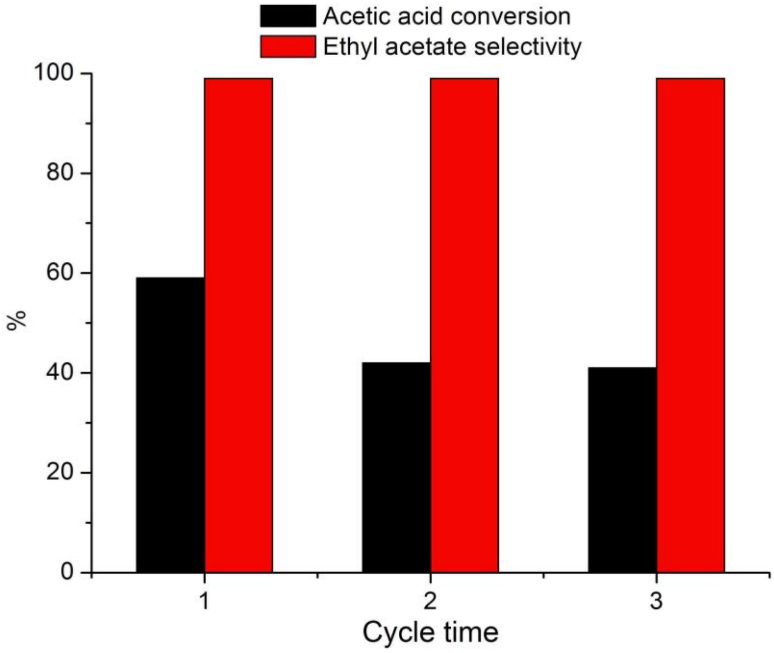

Supplementary Figure 11. Reusability of the H–Mo–Te oxide molecular wires for the esterification of acetic acid and ethanol.

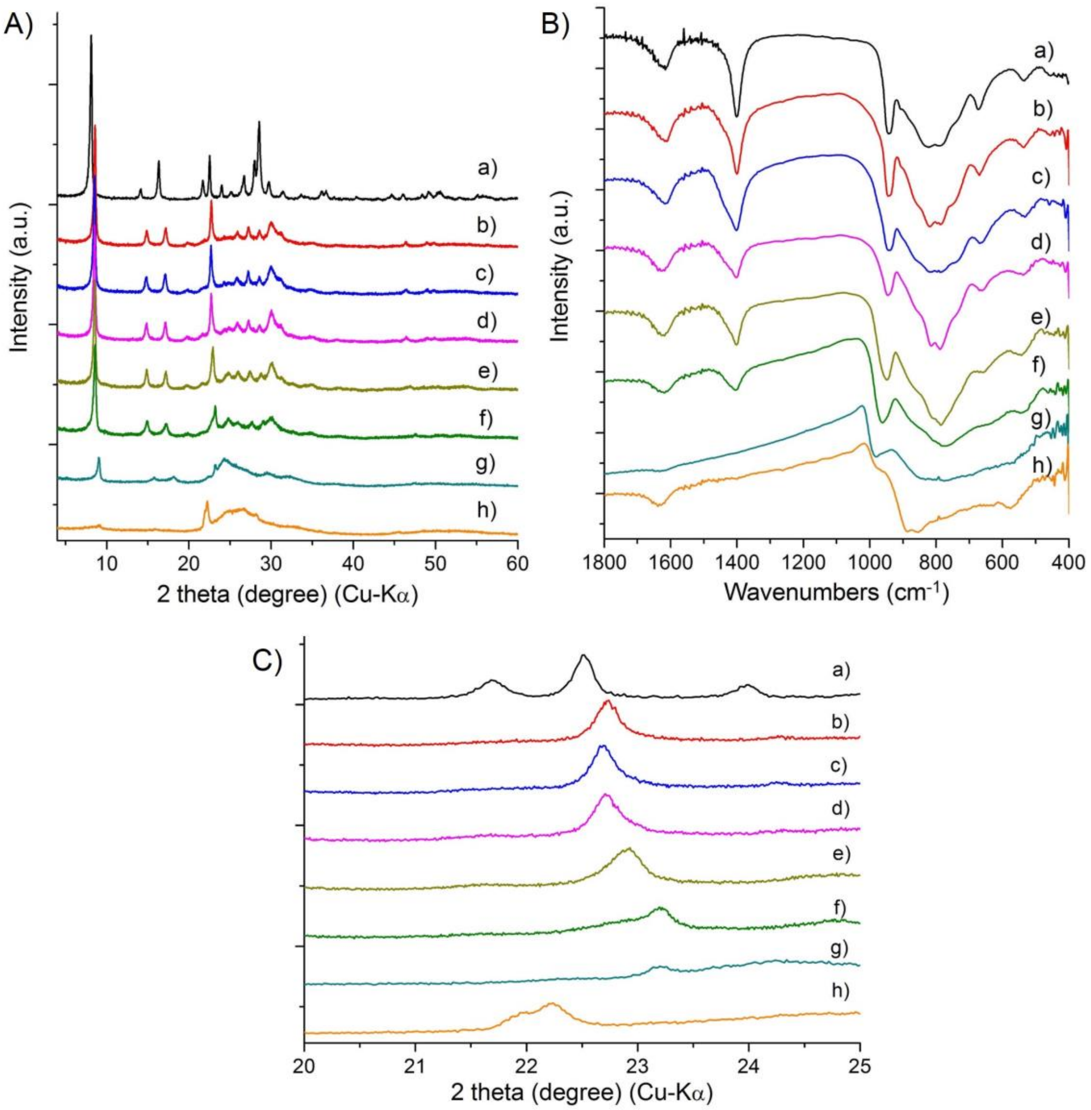

Supplementary Figure 12. XRD patterns. (A) and FT-IR spectra (B) of Mo–Te oxide a) as-synthesized, b) calcined at 473 K for 1 h, c) calcined at 498 K for 1 h, d) calcined at 523 K for 1 h, e) calcined at 548 K for 1 h, f) calcined at 573 K for 1 h, g) calcined at 623 K for 1 h, and h) calcined at 673 K for 1 h, and (C) magnified powder XRD patterns.

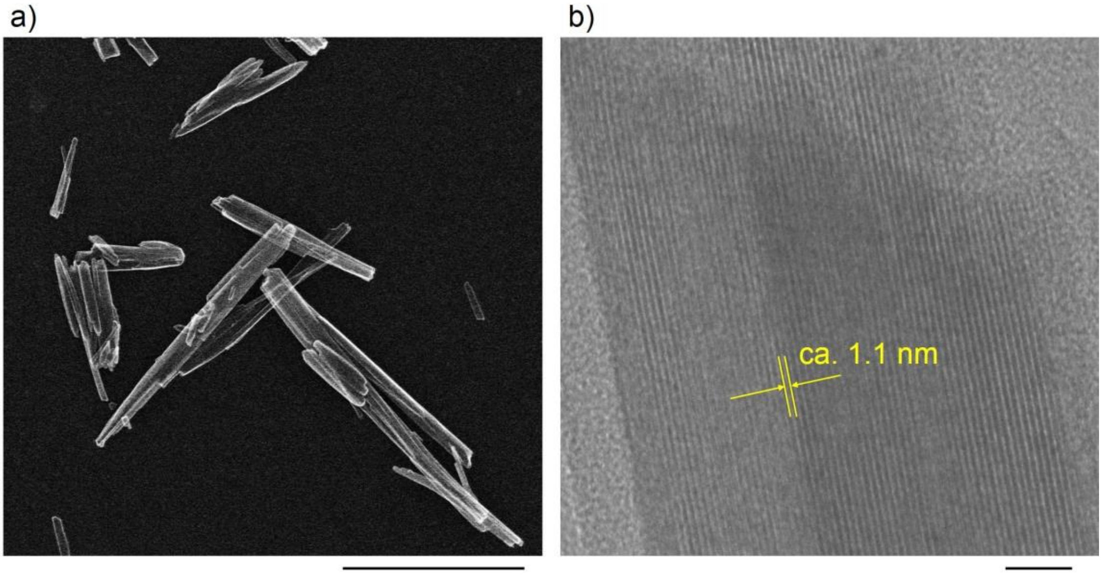

Supplementary Figure 13. Electron microscope images of calcined Mo–Te oxide at 573 K in N_2. a) SEM image; scale bar = 3 μm and b) TEM image; scale bar = 10 nm.

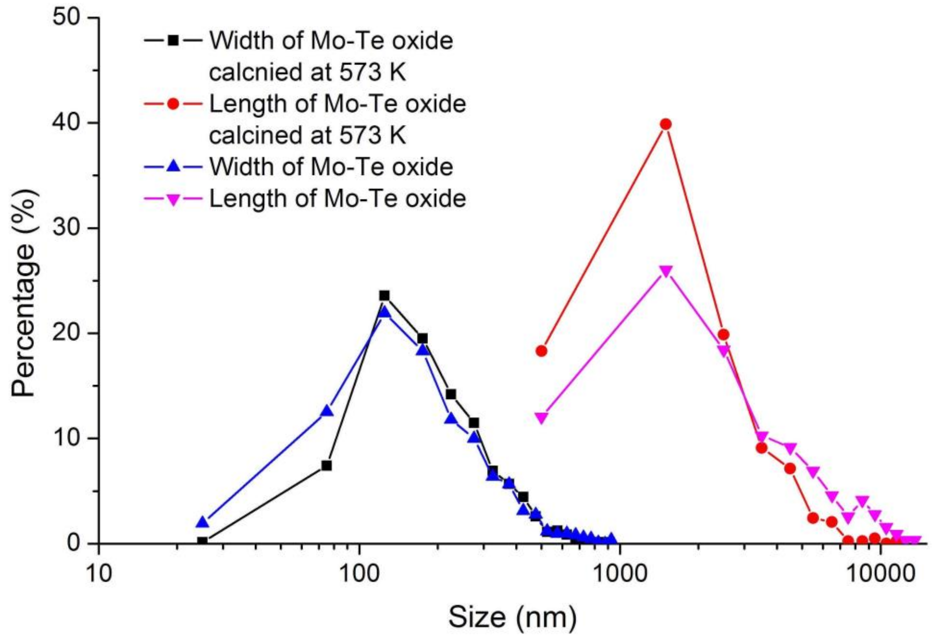

Supplementary Figure 14. Size distribution of the Mo–Te oxide crystal and the calcined Mo–Te oxide crystal at 573 K from the SEM images. More than 800 particles were counted.

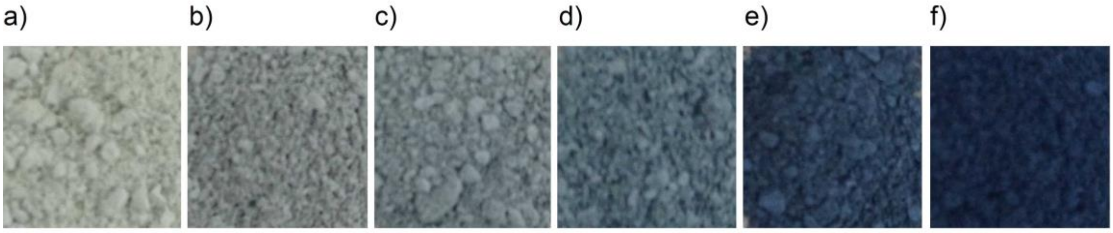

Supplementary Figure 15. **Photo images.** a) As-synthesized Mo–Te oxide, calcined under N_2 for 1 h at b) 473 K, c) 498 K, d) 523 K, e) 548 K, and f) 573 K.

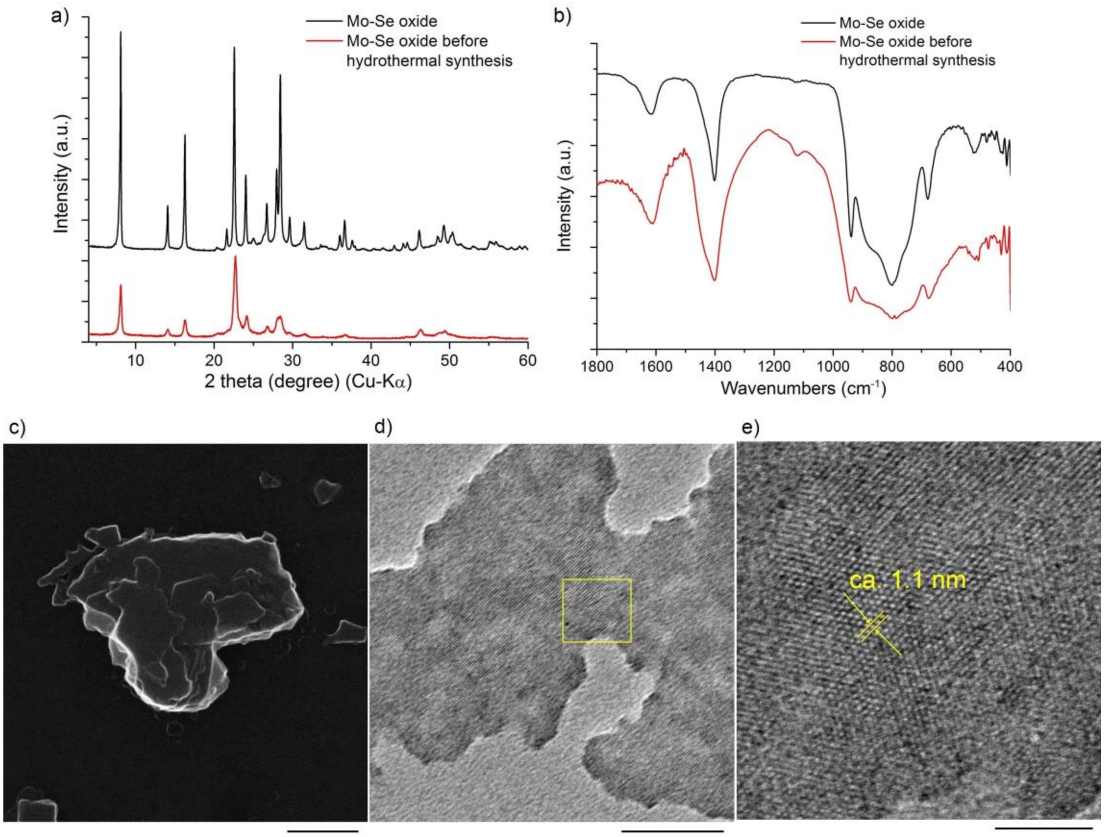

Supplementary Figure 16. Characterization of Mo–Se oxide obtained before hydrothermal synthesis. Comparison of a) XRD patterns and b) FT-IR spectra of Mo–Se oxide and Mo–Se oxide obtained before hydrothermal synthesis; c) SEM image, scale bar = 400 nm and d) TEM image of Mo–Se oxide obtained before hydrothermal synthesis, scale bar = 50 nm, and e) magnified TEM image in d) surrounded by yellow framework, scale bar = 20 nm.

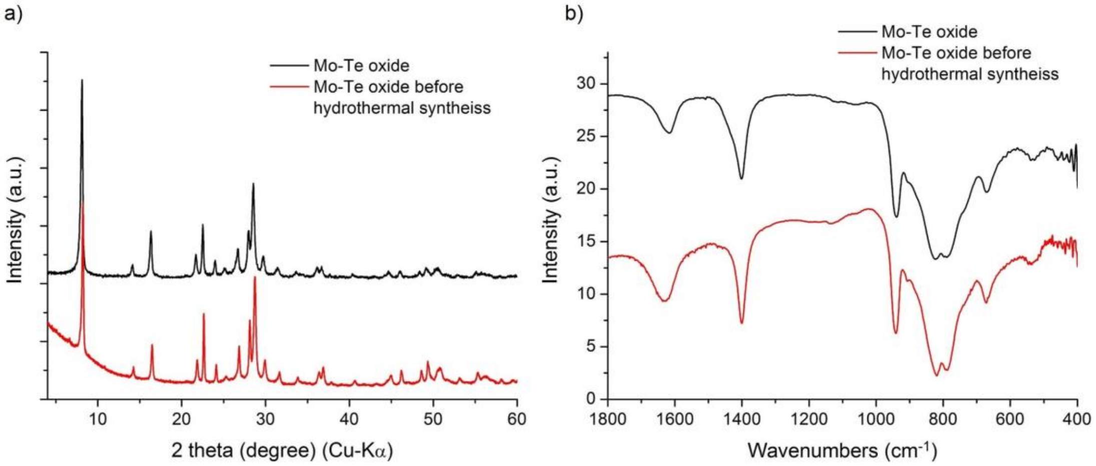

Supplementary Figure 17. Comparison of Mo–Te oxide before and after hydrothermal synthesis. a) Powder XRD patterns and b) FT-IR spectra.

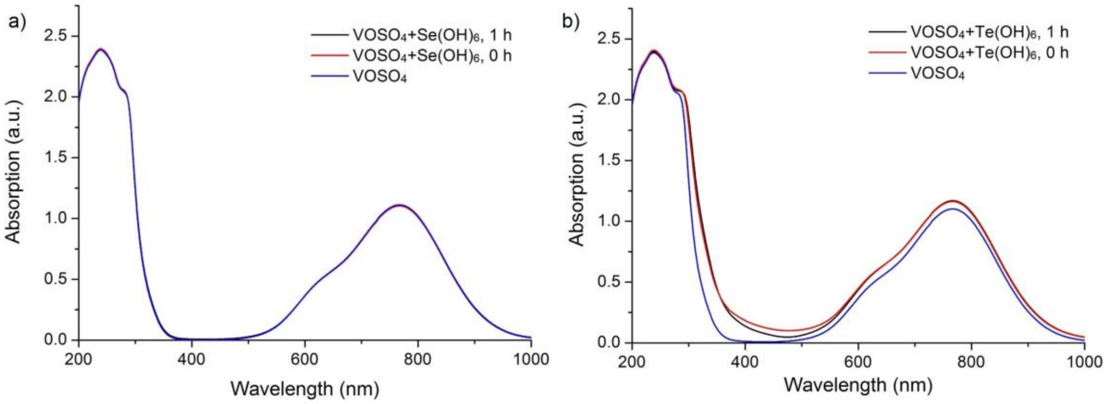

Supplementary Figure 18. UV-vis spectra. a) Black: $VOSO_4$ (0.0625 M)+$Se(OH)_6$ (0.0425 M) for 1 h, red: $VOSO_4$ (0.0625 M)+$Se(OH)_6$ (0.0425 M) for 0 h, and blue: $VOSO_4$ (0.0625 M) in water at room temperature and b) black: $VOSO_4$ (0.0625 M)+$Te(OH)_6$ (0.0425 M) for 1 h, red: $VOSO_4$ (0.0625 M)+$Te(OH)_6$ (0.0425 M) for 0 h, and blue: $VOSO_4$ (0.0625 M) in water at room temperature. UV-vis spectra showed almost no change after mixing $VOSO_4$ with $Te(OH)_6$ or $Se(OH)_6$, indicating that the reduction was slow at room temperature

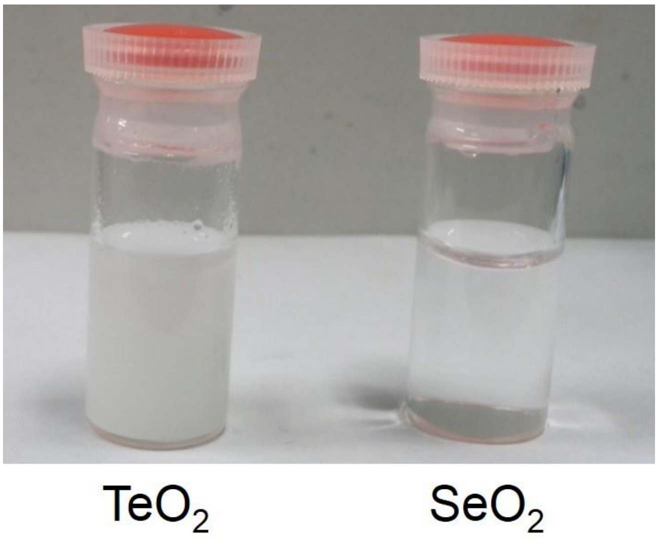

Supplementary Figure 19. **Photograph of TeO_2 and SeO_2 in water showing insoluble TeO_2 and soluble SeO_2.**

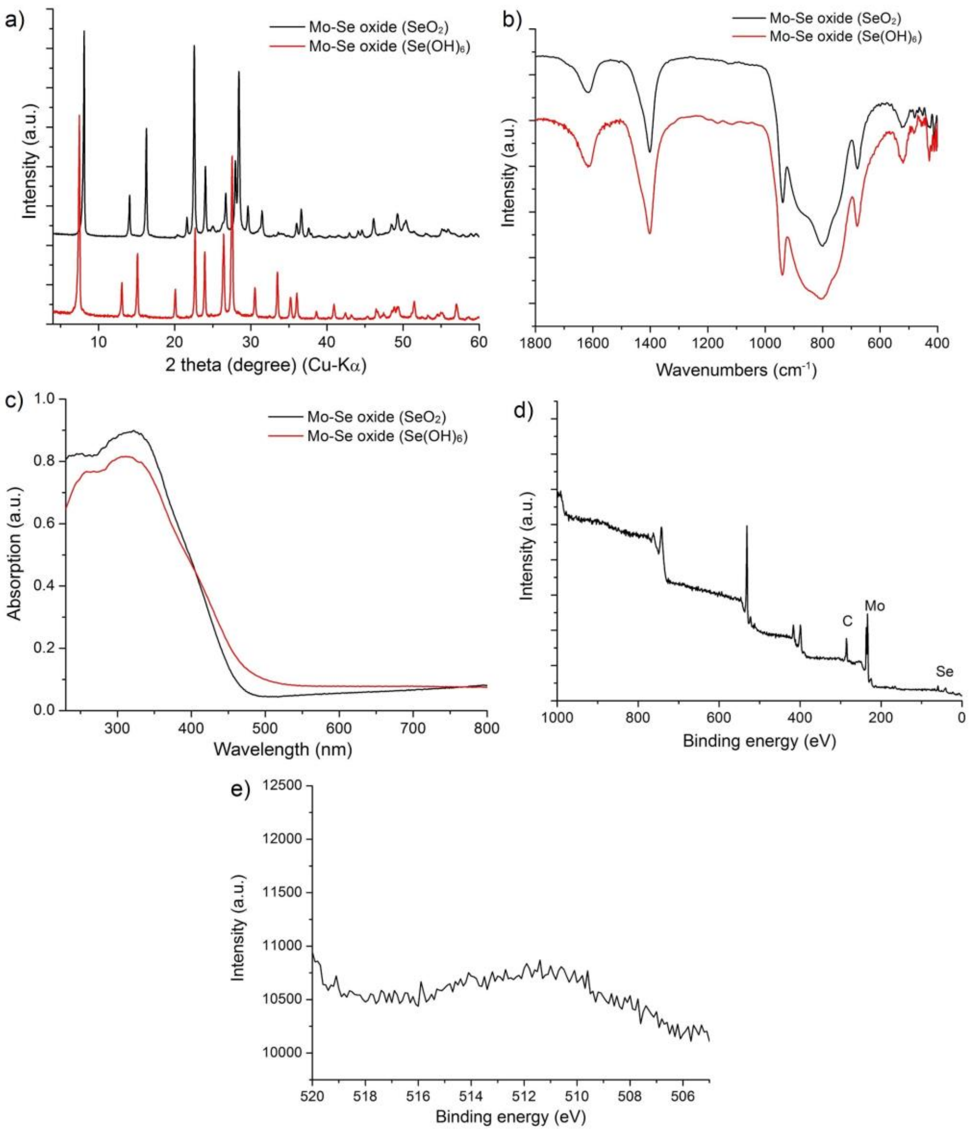

Supplementary Figure 20. Comparison of Mo–Se oxides obtained using SeO_2 or $Se(OH)_6$ and $VOSO_4$. a) Powder XRD patterns, b) FT-IR spectra, c) DR-UV-vis spectra, d) XPS profile at full-scale of Mo–Se oxide obtained using $Se(OH)_6$ + $VOSO_4$, and e) XPS profile in the range of $V(2p_{3/2})$, indicating that no vanadium was present in the material.

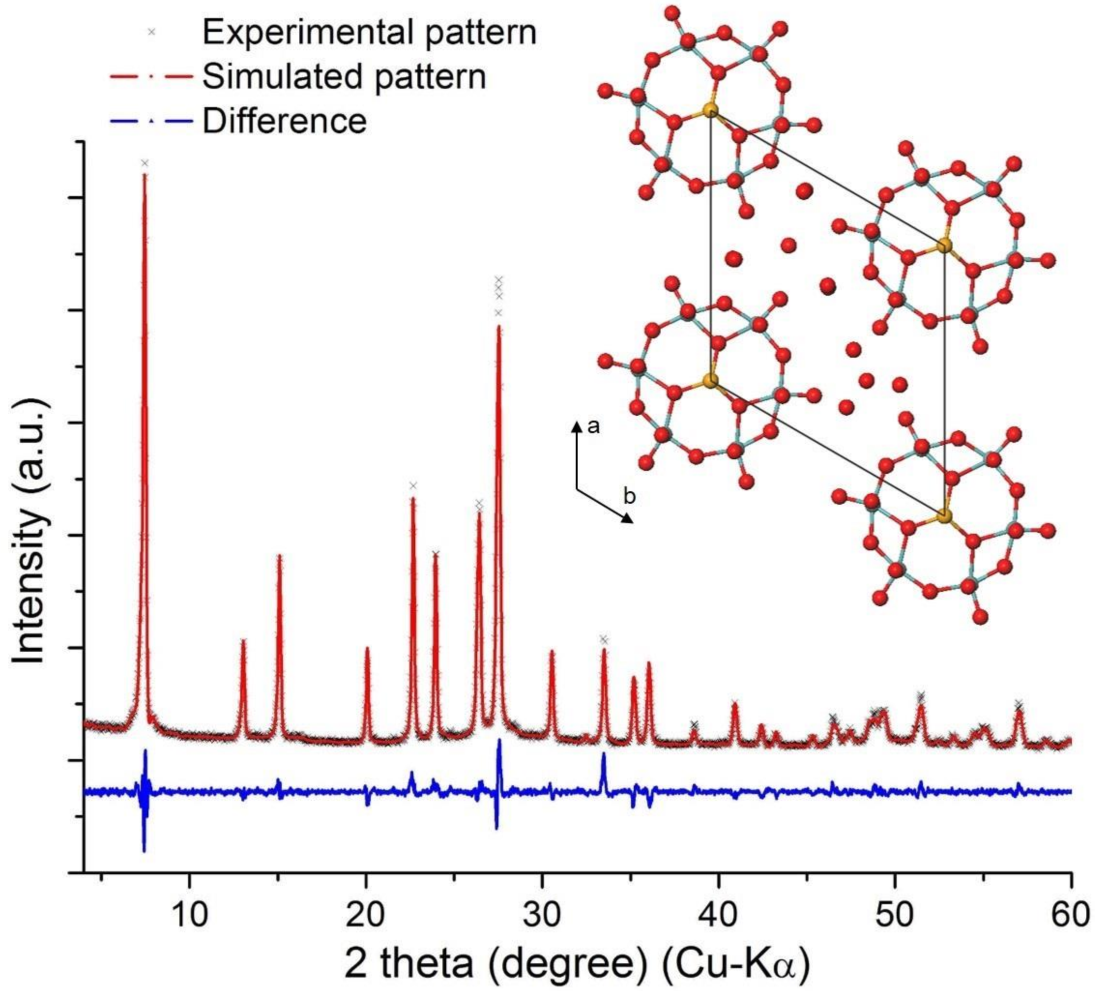

Supplementary Figure 21. Comparison of simulated powder XRD pattern of Mo–Se oxide obtained using $Se(OH)_6$ by Rietveld analysis with the experimental pattern. R_{wp} = 8.15%, insert: refined structure by the Rietveld method.

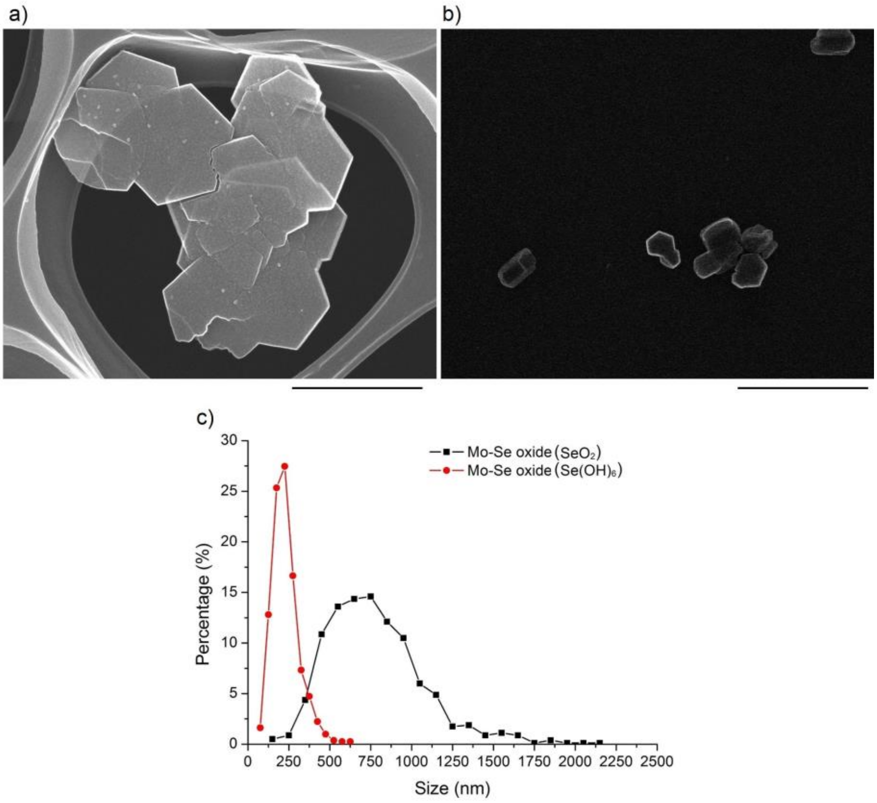

Supplementary Figure 22. Size comparison of Mo–Se oxides obtained using SeO_2 or $Se(OH)_6$ and $VOSO_4$. SEM images of a) Mo–Se oxide obtained using SeO_2, scale bar = 800 nm, b) Mo–Se oxide obtained using $Se(OH)_6$ and $VOSO_4$, scale bar = 800 nm, and c) size distribution based on the SEM images. Over 800 particles were counted.

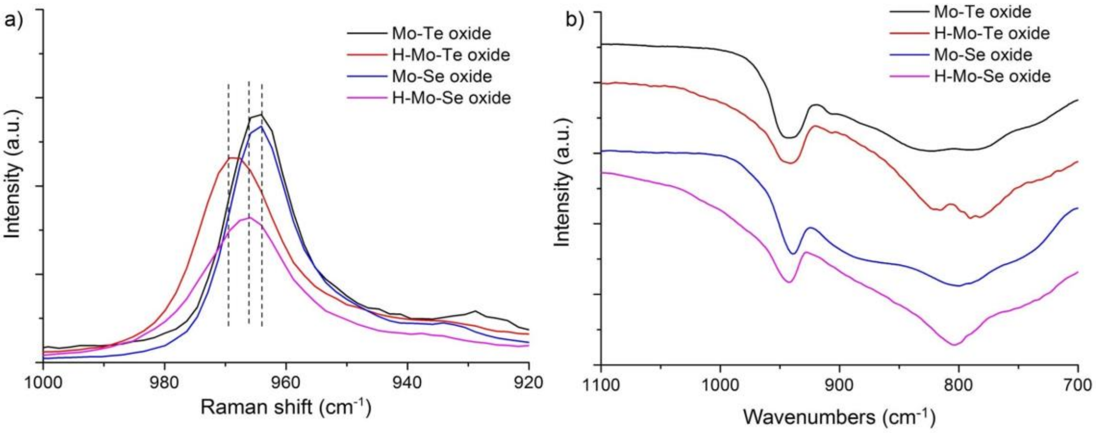

Supplementary Figure 23. Comparison of the as-synthesized materials with the proton-exchanged materials. a) Raman spectra and b) FT-IR spectra of Mo–Te oxide, H–Mo–Te oxide, Mo–Se oxide, and H–Mo–Se oxide

Supplementary Table 1. Crystallographic information and Rietveld refinement parameters for Mo–Te oxide and Mo–Se oxide obtained from powder XRD.

	Mo–Te oxide	Mo–Se oxide
crystal system	trigonal	trigonal
space group	*P*3	*P*3
$a = b$ (Å)	12.4824	12.5140
c (Å)	3.9366	3.9254
V ($Å^3$)	531.19	532.36
agreement factors		
R_{wp}	7.06%	7.49%
$R_{wp(w/o\ bck)}$	14.14%	15.31%
R_p	5.40%	5.48%
pattern parameter		
peak shape		
function	Pseudo-Voigt	Pseudo-Voigt
FWHM	U = 3.37442, V = -1.01679, W = 0.12166	U = 2.15759, V = -0.69664, W = 0.07751
profile parameter	N_A = 0.75970, N_B = 0.00267	N_A = 0.79328, N_B = 0.00229
line shift		
instrument geometry	Bragg-Brentano	Bragg-Brentano
zero point	-0.09029	-0.56883
shift#1	-0.06856	0.51637
shift#2	0.12100	0.13244
correction:		
method	Berar-Baldinozzi	Berar-Baldinozzi
parameter	P1 = 0.99732, P2 = 0.29346, P3 = -2.16470, P4 = -0.62694	P1 = 0.00756, P2 = 0.08260, P3 = -0.22348, P4 = -0.21355
background coefficients	polynomial = 100	polynomial = 100
preferred orientation		
function	March-Dollase R0 = 0.98137	March-Dollase R0 = 0.82412

Supplementary Table 2. Crystallographic data for Mo–Te oxide and Mo–Se oxide.

	Mo–Te oxide	Mo–Se oxide
method	single crystal analysis	powder X-ray diffraction
formula	$H_{14}N_2Mo_6O_{24}Te$	$Mo_6O_{24.3}Se$
*M*r	1129.37	1043.72
crystal system	trigonal	trigonal
space group	*P3*	*P3*
a (Å)	12.56(3)	12.5140
c (Å)	3.944(9)	3.9254
V (Å^3)	539(3)	532.36
T (K)	100(2)	298
Z	1	1
ρ_{calcd} (g·cm^{-3})	3.482	3.26
F_{000}	524	-
λ (Å)	0.78179	-
μ (mm^{-1})	6.182	-
measured reflections	2913	-
unique reflections	1171	-
$R1(I > 2\sigma(I))$	0.0812	-
w*R*2(all data)	0.2027	-
GOF	0.802	-

Supplementary Table 3. Structural information for Mo–Te oxide from single crystal analysis.

atom	x	Y	z	temperature factor (U)	occupancy
Te1A	0	0	0.922(3)	0.078(8)	0.47(3)
Te1B	0	0	0.474(3)	0.081(7)	0.53(3)
Mo1	-0.1661(9)	-0.3299(6)	0.1848(8)	0.121(4)	1
Mo2	0.1634(9)	-0.1620(8)	0.1863(8)	0.113(3)	1
O1	0.133(5)	-0.184(5)	0.710(14)	0.17(2)	1
O2	-0.132(4)	-0.294(4)	0.718(12)	0.103(13)	1
O3	-0.003(5)	-0.267(5)	0.228(14)	0.17(2)	1
O4	0.316(3)	-0.001(4)	0.203(12)	0.146(19)	1
O5	0.212(4)	-0.265(4)	0.211(10)	0.123(15)	1
O6	-0.210(6)	-0.479(6)	0.210(15)	0.18(2)	1
O7	0.139(3)	0.001(4)	1.198(10)	0.116(12)	1
O10	0.481(6)	-0.033(5)	0.72(2)	0.185(19)	1
H10A	0.4465	0.0131	0.613	0.278	1
H10B	0.4643	-0.0917	0.567	0.278	1
N11	0.3333	-0.3333	-0.21(2)	0.12(3)	1
H11A	0.254	-0.4151	-0.129	0.181	1
H11B	0.3333	-0.3333	-0.4215	0.181	1
N12	-0.3333	-0.6667	-0.272(14)	0.058(15)	1
H12A	-0.2747	-0.6026	-0.1939	0.087	1
H12B	-0.3333	-0.6667	-0.483	0.087	1

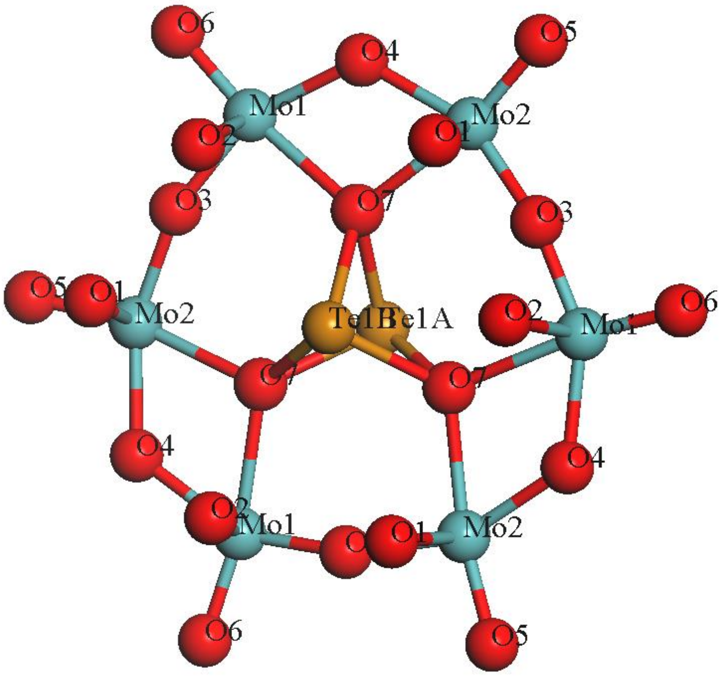

Supplementary Table 4. Bond lengths in Mo–Te oxide from single crystal analysis.

	bond length (Å)
Te1A-O7	2.05(4)
Te1B-O7	2.05(3)
Mo1-O6	1.67(6)
Mo1-O3	1.79(6)
Mo1-O2	1.90(5)
Mo1-O4	1.99(6)
Mo1-O2'	2.15(5)
Mo1-O7	2.24(4)
Mo2-O5	1.68(4)
Mo2-O3	1.84(5)
Mo2-O1	1.91(6)
Mo2-O4	1.97(4)
Mo2-O1'	2.09(5)
Mo2-O7	2.22(4)

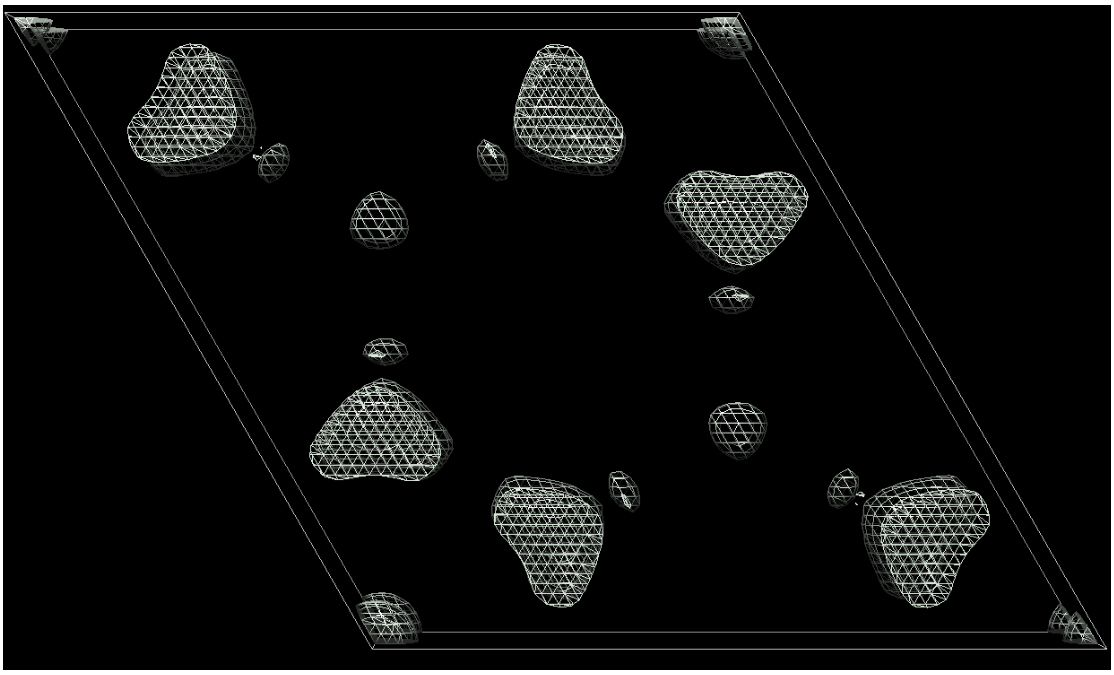

Supplementary Table 5. Results for Mo–Se oxide from the charge flipping method and peak assignments.

	x	y	z	intensity	assignment
1	0.3257	0.163	0.0316	51.82	Mo
2	0	0	0.2471	37.45	Se1
3	0	0	0.8169	35.87	Se2
4	0.332	0.6667	0.5332	12.17	O(cation or water)
5	0.4592	0.2274	0.0364	7.91	O(framework)
6	0.3111	-0.0009	0.0351	5.13	O(framework)
7	0.4607	0.4573	0.5252	3.31	O (cation or water)
8	0.1386	0.0686	0.0251	3.76	O(framework)
9	0.3081	0.155	0.6145	2.99	O(framework)
10	0.3198	0.1883	0.4522	3.11	-
11	0.1259	0.8109	0.46	2.76	-
12	0.3767	-0.0028	0.5321	2.57	-

Supplementary Table 6. Structural information for Mo–Se oxide from Rietveld analysis.

atom	x	y	z	U_{iso}	occupancy
Mo2	0.32358	0.13747	0.2359	0.01	1
O1	0.31892	0.15665	-0.27955	0.06	1
O3	0.47793	0.23633	0.24878	0.06	1
O5	0.28615	-0.01236	0.21169	0.06	1
Mo3	-0.3166	-0.15994	0.15314	0.01	1
O2	-0.32084	-0.15273	-0.35192	0.06	1
O4	-0.46363	-0.20989	0.24878	0.06	1
O6	-0.27077	0.01449	0.20205	0.06	1
O7	-0.15387	-0.16077	0.23457	0.06	1
O8	0.49977	-0.00198	0.80953	0.06	0.5
O9	0.33333	0.66667	0.93032	0.06	0.91
O10	0.66667	0.33333	0.80478	0.06	0.91
Se1	0	0	0.40341	0.02	1

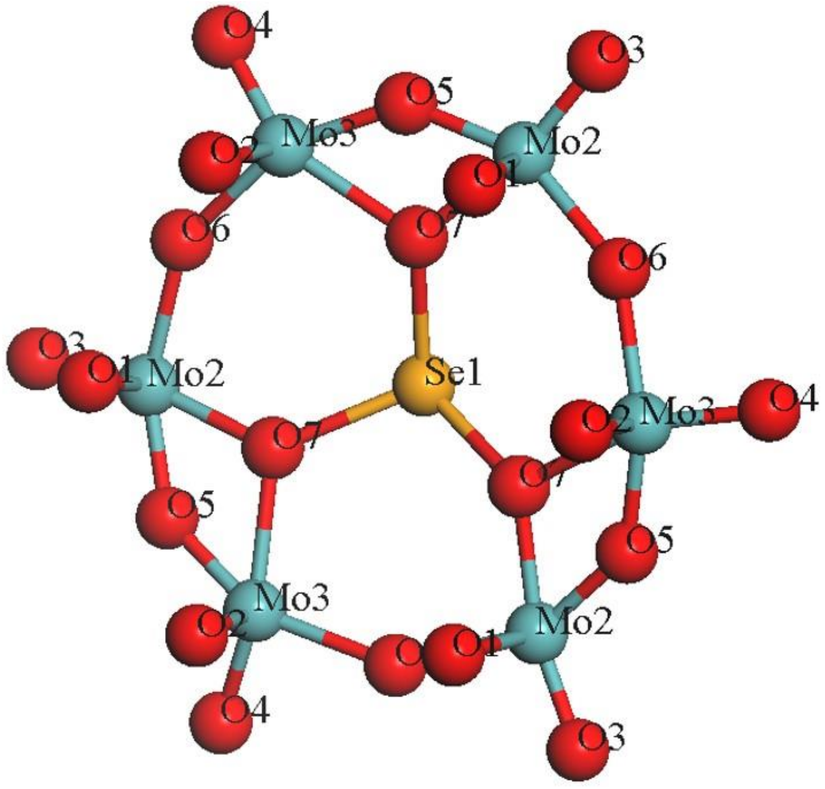

Supplementary Table 7. Bond lengths in Mo–Se oxide from Rietveld analysis.

	bond length (Å)
Mo2-O3	1.695
Mo2-O5	1.693
Mo2-O1	2.042
Mo2-O6	1.957
Mo2-O7	1.869
Mo2-O1’	1.922
Mo3-O4	1.663
Mo3-O6	1.969
Mo3-O2	1.987
Mo3-O5	1.719
Mo3-O2’	1.947
Mo3-O7	2.067
Se1-O7	2.079

Supplementary Methods

Synthesis and characterization of Mo–Se oxide before hydrothermal synthesis.

$(NH_4)_6Mo_7O_{24}\cdot4H_2O$ (1.766 g, 10 mmol based on Mo) was dissolved in 20 mL of water, followed by addition of SeO_2 (0.189 g, 1.7 mmol) to the $(NH_4)_6Mo_7O_{24}\cdot4H_2O$ solution. The pH of the solution was adjusted to 2.8 by H_2SO_4 (1 M), and the solution was stirred at room temperature for 10 min (yellow solid was generated during this process) and degassed by N_2 bubbling for 10 min. The solid was collected by filtration, washed with water (10 mL) 3 times, and dried at room temperature overnight. Then, 0.18 g of Mo–Se oxide was obtained (yield of 10.2 %).

XRD patterns and FT-IR spectra (Supplementary Figure 16) showed that the structure of Mo–Se oxide before hydrothermal synthesis was identical to that after hydrothermal synthesis. However, the crystallinity of Mo–Se oxide before hydrothermal synthesis was low. SEM (Supplementary Figure 16) showed an irregular-shapped particle, but a lattice image was observed in TEM. Mo–Se oxide was produced immediately after mixing of the Mo and Se sources; the morphology was undefined in shaped, and the crystallinity was lower than the Mo–Se oxide obtained after hydrothermal reaction. Under hydrothermal conditions, crystallinity increased, and the crystal grew to a hexagonal plate-shape.

Synthesis and characterization of Mo–Te oxide before hydrothermal synthesis.

$(NH_4)_6Mo_7O_{24}\cdot4H_2O$ (1.766 g, 10 mmol based on Mo) was dissolved in 20 mL of water, followed by addition of $Te(OH)_6$ (0.391 g, 1.7 mmol) to the $(NH_4)_6Mo_7O_{24}\cdot4H_2O$ solution to form solution A. Then, $VOSO_4\cdot5H_2O$ (0.6438 g, 2.54 mmol) was dissolved in 20 mL of water to form solution B. Solution B was poured rapidly into solution A. The mixture was stirred at room temperature for 10 min and degassed by N_2 bubbling for 10 min (pH 2.8). The solution was sealed and stored at room temperature for 2 days. Less than 1 mg of Mo–Te oxide was obtained (yield $<$ 0.06% based on Mo).

After the precursor solution was prepared, no precipitate was observed within one day, indicating that the crystal growth was slow. After approximately 2 days, a small amount of solid ($<$1 mg) was recovered, which was characterized by XRD and FT-IR analysis and indicated that the structure of the material was identical to that of the material after hydrothermal synthesis (Supplementary Figure 17). These results demonstrate that the formation and crystallization of Mo–Te oxide is slow.

Synthesis of Mo–Se oxide using $Se(OH)_6$ and $VOSO_4$.

$(NH_4)_6Mo_7O_{24}\cdot4H_2O$ (1.766 g, 10 mmol based on Mo) was dissolved in 20 mL of water, followed by addition of $Se(OH)_6$ (0.308 g, 1.7 mmol) to the $(NH_4)_6Mo_7O_{24}\cdot4H_2O$ solution to form solution A. Then, $VOSO_4\cdot5H_2O$ (0.6438 g, 2.5 mmol) was dissolved in 20 mL of water to form solution B. Solution B was poured rapidly into solution A. The mixture was stirred at room temperature for 10 min and degassed by N_2 bubbling for 10 min (pH~2.8). The mixture was introduced into the 50-mL Teflon-liner of a stainless-steel autoclave, which was heated at 448 K for 24 h. After the autoclave had been cooled to room temperature, the resulting solid was recovered from the solution by filtration. The obtained solid was washed with 10 mL of water 3

times and dried at 353 K overnight. Then, 0.3 g of Mo–Se oxide was obtained (yield of 17% based on Mo).
